# Explaining Support Vector Machines: A Color Based Nomogram

**DOI:** 10.1371/journal.pone.0164568

**Published:** 2016-10-10

**Authors:** Vanya Van Belle, Ben Van Calster, Sabine Van Huffel, Johan A. K. Suykens, Paulo Lisboa

**Affiliations:** 1 Department of Electrical Engineering (ESAT), STADIUS Center for Dynamical Systems, Signal Processing and Data Analytics, KU Leuven, Leuven, Belgium; 2 iMinds Medical IT, Leuven, Belgium; 3 Department of Development and Regeneration, KU Leuven, Leuven, Belgium; 4 Department of Applied Mathematics, Liverpool John Moores University, Liverpool, United Kingdom; Roswell Park Cancer Institute, UNITED STATES

## Abstract

**Problem setting:**

Support vector machines (SVMs) are very popular tools for classification, regression and other problems. Due to the large choice of kernels they can be applied with, a large variety of data can be analysed using these tools. Machine learning thanks its popularity to the good performance of the resulting models. However, interpreting the models is far from obvious, especially when non-linear kernels are used. Hence, the methods are used as black boxes. As a consequence, the use of SVMs is less supported in areas where interpretability is important and where people are held responsible for the decisions made by models.

**Objective:**

In this work, we investigate whether SVMs using linear, polynomial and RBF kernels can be explained such that interpretations for model-based decisions can be provided. We further indicate when SVMs can be explained and in which situations interpretation of SVMs is (hitherto) not possible. Here, explainability is defined as the ability to produce the final decision based on a sum of contributions which depend on one single or at most two input variables.

**Results:**

Our experiments on simulated and real-life data show that explainability of an SVM depends on the chosen parameter values (degree of polynomial kernel, width of RBF kernel and regularization constant). When several combinations of parameter values yield the same cross-validation performance, combinations with a lower polynomial degree or a larger kernel width have a higher chance of being explainable.

**Conclusions:**

This work summarizes SVM classifiers obtained with linear, polynomial and RBF kernels in a single plot. Linear and polynomial kernels up to the second degree are represented exactly. For other kernels an indication of the reliability of the approximation is presented. The complete methodology is available as an R package and two apps and a movie are provided to illustrate the possibilities offered by the method.

## Introduction

Support vector machines (SVMs) have proven to be good classifiers in all kinds of domains, including text classification [1], handwritten digit recognition [2], face recognition [3], bioinformatics [4], among many others. Thanks to the large variety of possible kernels, the application areas of SVMs are widespread. However, although these methods generalize well to unseen data, decisions made based on non-linear SVM predictions are difficult to explain and as such are treated as black boxes. For clinical applications, information on how the risk of disease is estimated from the inputs is crucial information to decide upon the optimal treatment strategy and to inform patients. Being able to discuss this information with patients might enable patients to change their behaviour, life style or therapy compliance. Interpretation is especially important for validation of the model inferences by subject area experts. The fact that machine learning techniques are not able to find their way into clinical practice might very well be related to the lack of acquiring this information.

Offering interpretation to SVMs is a topic of research with different perspectives [5]. Identification of prototypes [6] (interpretability in dual space) offers an interpretation closely related to how doctors work: based on experience from previous patients (the prototypes) a decision is made for the current patient. A second view on interpretability (interpretability in the input space) intends to offer insights in how each input variable influences the decision. Some researchers worked on a combination of both [7] and identified prototypes dividing the input space into Voronoi sections, within which a linear decision boundary is created, offering interpretation w.r.t. the effect of the inputs in a local way. Other approaches try to visualize the decision boundary in a two-dimensional plane [8], using techniques related to self-organizing maps [9]. The current work attempts to offer a global interpretation in the input space.

The literature describes several methods to extract rules from the SVM model (see [10, 11] and references therein) in order to provide some interpretation of the decisions obtained from SVM classifiers. However these rules do not always yield user-friendly results, and when inputs are present in several rules, identifying how the decision will change depending on the value of an input is not straightforward.

Several authors have therefore tried to open the black box by attempts to visualize the effect of individual inputs to the output of the SVM. In [12], Principal Component Analysis is used on the kernel matrix. Biplots are used to visualize along which principal components the class separability is the largest. To visualize which original inputs contribute the most to the classifier, pseudosamples with only one input differing from zero are used to mark trajectories within the plane spanned by the two principal components identified before. Those inputs with the largest trajectories along the direction of largest class separability are the most important inputs. Although this approach enables to visualize which inputs are most relevant, it is not possible to indicate how the output of the classifier (i.e. the latent variable or the estimated probability) would change in case the value of one input would change.

A second method to visualize and interpret SVMs was proposed by [13] for support vector regression. They propose to multiply the input matrix containing the inputs of all support vectors with the Lagrange multipliers to get the impact of each input. This approach is again able to identify the most important inputs, but is not able to indicate how the output of the SVM changes with changing inputs.

Other work consists in visualizing the discrimination of data cohorts by means of projections guided by paths through the data (tours) [14–16]. Although these methods offer additional insights, they do not quantify the impact of each feature on the prediction, which is the goal of the current work.

**Fig 1:**
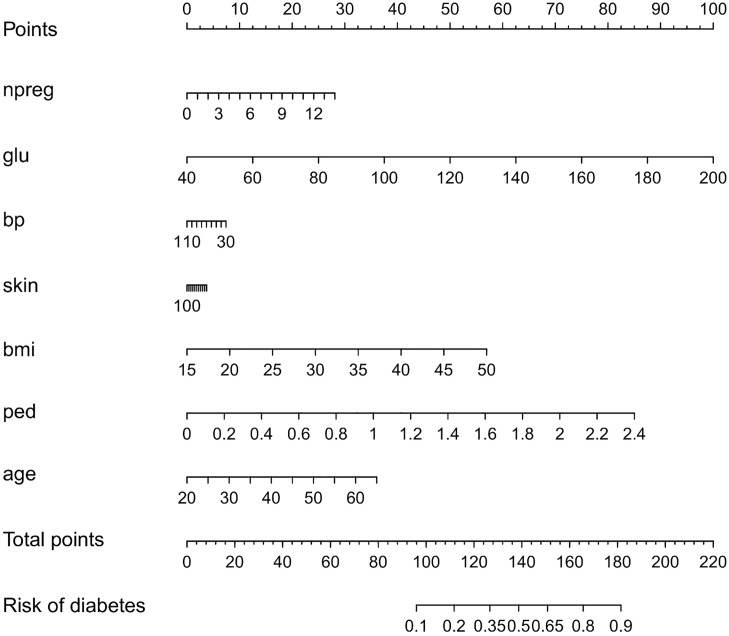
Visualization of the logistic regression model for the Pima dataset by means of a nomogram. The contribution of each input variable *x*^(*p*)^ (*f*^(*p*)^ = *w*^(*p*)^ *x*^(*p*)^) to the linear predictor is shifted and rescaled such that each contribution has a minimal value of zero and the maximal value of all contributions is 100. Each input variable is represented by means of a scale and the value of the contribution can be found by drawing a vertical line from the input variable value to the points scale on top of the plot. Adding the contributions of all input variables results in the total points. These can be transformed into a risk estimate by drawing a vertical line from the total points scale to the risk scale. The importance of the inputs is represented by means of the length of the scales: variables with longer scales have a larger impact on the risk prediction.

Standard statistical methods such as linear and logistic regression offer the advantage that they are interpretable in the sense that it is clear how a change in the value of one input variable will affect the predicted outcome. To further clarify the impact of the input variables, visualization techniques such as nomograms [17] can be used (see Fig 1). In short, a nomogram represents a linear model $\hat{y}=\sum_{p=1}^{d}\;w^{\left(p\right)}x^{\left(p\right)}+b$, with *x*^(*p*)^ the *p*^th^ input and *w*^(*p*)^ the corresponding weight, by means of lines, the length of which is related to the range of *w*^(*p*)^ *x*^(*p*)^ observed in the training data. For each input value the contribution to the predicted outcome can instantly be read of from the plot. See Section Logistic regression models for more information. Straightforward extension of this technique to SVMs is not possible due to the fact that the SVMs are mainly used in combination with flexible kernels that can not be decomposed into additive terms, each accounting for one single input variable. A possible extension of nomograms towards support vector machines [18] therefore focusses on the use of decomposeble kernels [19]. The most restricting way of applying this approach is to define a kernel as the addition of subkernels that only depend on one single input. The use of a localized radial basis function kernel in [20] is only one example. The original work of [18] to represent SVMs by means of nomograms is less restrictive in the sense that kernels including interactions between two inputs are allowed. The idea behind these approaches is that by using a decomposible kernel, the latent variable of the SVM can be expressed as a sum of terms, depending on one input. As such, the SVM becomes a generalized additive model and can be visualized by means of a nomogram. In [18] non-linearities are visualized by drawing two-dimensional curves instead of straight lines in the nomogram, such that non-linearities can more easily be represented than when using a line. They also allow for interactions between two inputs, but, as with standard nomograms, they can only be represented after categorization of one of the two involved inputs.

In contrast with the approaches found in the literature, this work does not intend to adapt the kernel nor the SVM model formulation. This work takes the first steps in answering the question whether existing SVMs in combination with generally used kernels can be explained and visualized, in which circumstances this is possible and to which extent. Instead of adapting the kernel, the nomogram representation is altered to easily allow for non-linear and two-way interaction effects. This is achieved by replacing the lines by color bars with colors offering the same interpretation as the length of the lines in nomograms. It is indicated for which kernels and kernel parameters the representation by means of this color based nomogram is exact. In cases where the visualization is only approximate, additional graphs indicate why the approximation is not sufficient and how this might be solved. The current approach is related to the work in [21, 22], where a Taylor expansion of the RBF kernel is used to extract interpretable and visualizable components from an SVM with RBF kernel. In this work, the expansion is indicated for linear, polynomial and RBF kernels. Additionally, the expansion is used to visualize the working of an existing SVM, whereas in the previous work a new model was created after feature selection by means of iterative *l*_1_ regularization of a parametric model with the different components as inputs.

The remainder of this work is structured as follows. First, a short introduction to SVM classification is given. It is shown how a nomogram is built for logistic regression models and how an alternative color based nomogram for logistic regression was used in [23]. Next, it is explained how to reformulate the SVM classifier in the same framework. Experiments on artificial data illustrates the approach and indicates possible problems and solutions. Finally, real life datasets are used to illustrate the applicability on real examples. The work concludes with information on the available software and a discussion on the strengths and weaknesses of the study.

## Methods

This section clarifies how an SVM can be explained by means of a color based nomogram. For generality, we start with a brief summary of an SVM classifier, followed by an introduction on the use of a nomogram to visualize logistic regression models.

In the remainder of this work, ${x_{i}^{\left(p\right)}}^{m}$ will indicate the *m*^th^ power of the *p*^th^ input variable of the *i*^th^ observation *x*_*i*_.

### SVM classifier

Suppose a dataset $D={\left\{x_{i},y_{i}\right\}}_{i=1}^{N}$ is a set of *N* observations with input variables $x_{i}\in R^{d}$ and class labels *y*_*i*_ ∈ {−1, 1}. The SVM classifier as defined by Vapnik [24] is formulated as

$$\begin{array}{cc} \min_{w,b,\epsilon } & \frac{1}{2}w^{T}w+C\sum_{i=1}^{N}\epsilon_{i}\\ \text{subject}\;\text{to} & \left\{\begin{array}{c} \begin{array}{cc} y_{i}\left(w^{T}\varphi \left(x_{i}\right)+b\right)\geq 1-\epsilon_{i}, & \;\forall \;i=1,\ldots ,N\\ \epsilon_{i}\geq 0, & \;\forall \;i=1,\ldots ,N\;. \end{array} \end{array}\right. \end{array}$$ (1)

To facilitate the classification, a feature map *φ*(⋅) is used to transform the inputs into a higher dimensional feature space. The coefficients in this higher dimensional feature space are denoted by $w\in R^{n_{\varphi }}$. The trade-off between a smooth decision boundary and correct classification of the training data is made by means of the strictly positive regularization constant *C*.

The dual formulation of the problem stated in Eq (1) is found by defining the Lagrangian and characterizing the saddle point and results in:

$$\begin{array}{cc} \min_{\alpha } & \frac{1}{2}\sum_{i,j=1}^{N}y_{i}y_{j}\varphi {\left(x_{i}\right)}^{T}\varphi \left(x_{j}\right)\alpha_{i}\alpha_{j}-\sum_{i=1}^{N}\alpha_{i}\\ \text{subject}\;\text{to} & \left\{\begin{array}{c} \begin{array}{cc} \sum_{i=1}^{N}\alpha_{i}y_{i}=0\\ 0\leq \alpha_{i}\leq C, & \;\forall \;i=1,\ldots ,N\;. \end{array} \end{array}\right. \end{array}$$ (2)

The power of SVMs lies in the fact that the feature map does not need to be defined explicitly. An appropriate choice of a kernel function *K*(*x*, *z*) for any two points *x* and *z* that can be expressed as

$$K\left(x,z\right)=\varphi {\left(x\right)}^{T}\varphi \left(z\right)\;,$$

makes it possible to use an implicit feature map.

A class label for a new point *x* can then be predicted as

$$\hat{y}=\mathrm{sign}\left(\sum_{i=1}^{N}\alpha_{i}y_{i}K\left(x_{i},x\right)+b\right)\;.$$

Here $\ell =\sum_{i=1}^{N}\alpha_{i}y_{i}K\left(x_{i},x\right)+b$ is called the latent variable. In order to obtain probabilities, the sign(·) function can be replaced by a function *h*(⋅). In this work the latent variable will be converted into a risk estimate by using it as a single input in a logistic regression model with two parameters. This approach is known as Platt’s rule [25, 26].

### Visualization of risk prediction models

#### Logistic regression models

In statistics, regression models can be visualized using nomograms [17]. More recently color plots have been proposed [23] to represent contributions to the linear predictor (here $\sum_{p=1}^{d}\;w^{\left(p\right)}x^{\left(p\right)}+b$) depending on only one or by extension maximally two input variables. The nomogram for logistic regression (LR) builds on the fact that the model in its most basic form can be written as

$$\hat{p}=h\left(\sum_{p=1}^{d}w^{\left(p\right)}x^{\left(p\right)}+b\right)\;,$$ (3)

where *h*(⋅) is a link function (here the sigmoid function) transforming the linear predictor to a chance, *w*^(*p*)^ is the coefficient corresponding to the *p*^th^ input variable *x*^(*p*)^ and *b* is a constant. The contribution of each input variable *x*^(*p*)^ to the linear predictor can thus be visualized by plotting *f*^(*p*)^(*x*^(*p*)^) = *w*^(*p*)^ *x*^(*p*)^. In fact, for nomograms these terms are rescaled to start from 0 to a maximum of 100 points. Doing so makes clear that the range of the contributions is important. A wide range of the contributions for one input variable indicates that changing the value of this input, can have a large impact on the linear predictor and as such on the risk estimate. Fig 1 clarifies this approach for a logistic regression model trained on the Pima Indian diabetes dataset from the UCI repository [27]. The training data as provided in the R package MASS [28, 29] was used to train the logistic regression model. The nomogram was generated using the rms package [28, 30]. To obtain the risk estimate for an observation, the points corresponding to each input variable are obtained by drawing a vertical line from this value up to the points scale on top of the plot. These points are added to obtain the total points, which are converted to a risk by means of the bottom two scales.

**Fig 2:**
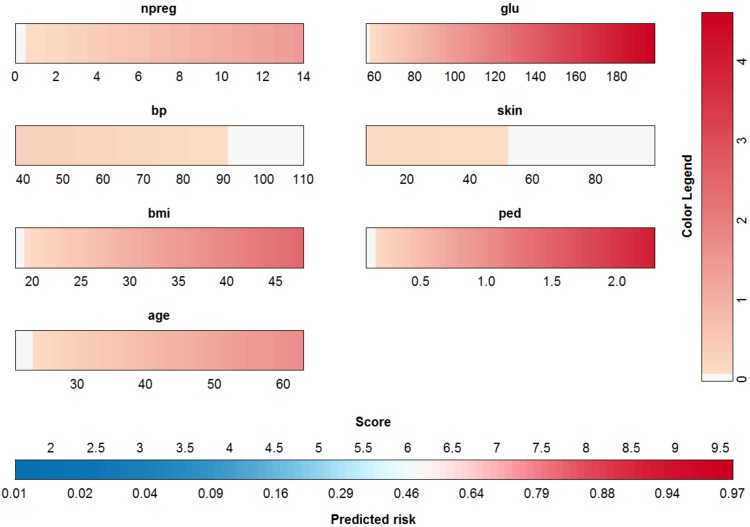
Visualization of the logistic regression model for the Pima dataset by means of a color plot or color based nomogram. The contribution of each input variable *x*^(*p*)^ (*f*^(*p*)^ = *w*^(*p*)^ *x*^(*p*)^) to the linear predictor is shifted such that each contribution has a minimal value of zero. To obtain a risk estimate for an observation, the color corresponding to the input’s value needs to be indicated. This color is converted to a point by means of the color legend at the right. Repeating this for each input and summing the resulting points, yields the score. This score is then converted into the risk estimate by means of the bottom most color bar. The importance of the inputs is represented by means of the redness of the color: variables with a higher intensity in red have a larger impact on the risk prediction.

A similar approach using the methods proposed in [23] is illustrated in Fig 2. Instead of scales, color bars are used, the color of which indicates the contribution of the input variable value. In this case, the contributions are shifted to make sure that the minimal contribution of each input is zero. The contributions are not rescaled. The importance of the inputs is clear from the color. The intenser the red color becomes within the color bar, the more impact this input has (similar to the length of the scales in the nomogram). To obtain a risk estimate for an observation, the procedure is as follows. For each input, find the color corresponding to the input’s value. This color is converted to a point by means of the color legend at the right. Repeating this for each input and summing the resulting points, yields the score. This score is then converted into the risk estimate by means of the bottom most color bar. A more detailed explanation of how this color based nomogram is constructed from the risk prediction model is given in S1 Text.

From both approaches (nomogram and color-based plot) it is easily concluded that glucose, the pedigree function and bmi are the most influential inputs.

#### Support vector classifiers

Whether or not an SVM classifier can be interpreted in the same way as explained above and represented by similar graphs, depends on the choice of the kernel. A derivation for the linear, polynomial and RBF kernel is given here.

When using a linear kernel $K_{\text{lin}}\left(x,z\right)=\sum_{p=1}^{d}\;x^{\left(p\right)}z^{\left(p\right)}$, the extension of the nomogram to an SVM is easily made. The predicted risk is found as:

$$\hat{y}=h\left(\sum_{i=1}^{N}\alpha_{i}y_{i}K\left(x_{i},x\right)+b\right)=h\left(\sum_{i=1}^{N}\alpha_{i}y_{i}\sum_{p=1}^{d}x_{i}^{\left(p\right)}x^{\left(p\right)}+b\right)\;,$$

such that the contribution of the *p*^th^ input variable to the linear predictor is defined as

$$f^{\left(p\right)}=\sum_{i=1}^{N}\alpha_{i}y_{i}x_{i}^{\left(p\right)}x^{\left(p\right)}\;.$$

This expansion enables to visualize an SVM model with a linear kernel using plots of the type presented in Fig 2. Each contribution *f*^(*p*)^ is then represented by a color bar. The points that are allocated to the value of an input are read off by means of the color legend. The score is obtained by addition of all points. The function *h*(⋅) converting this score to a risk estimate is visualized by another color bar at the bottom of the graph. Examples of this type of representation for SVM models are given in Section Results.

This approach can also be extended to other additive kernels [31] and ANOVA kernels [19, 24], in which kernels are expressed as a sum of subkernels, each of which depend on a restricted set of input variables. In cases were no more than two inputs are involved in each subkernel, the representation will be exact. Visualization of two-way interaction effects is done by the use of color plots instead of color bars. Examples of this approach are given in Section Results.

For the polynomial kernel *K*_poly_(*x*, *z*) = (*ax*^*T*^ *z* + *c*)^*δ*^, with *δ* a positive integer, an expansion of the latent variable is found by use of the multinomial theorem [32]

$${\left(x^{\left(1\right)}+\cdots +x^{\left(d\right)}\right)}^{\delta }=\sum_{k_{1}+\cdots +k_{d}=\delta }\left(\begin{array}{c} \delta \\ k_{1},\ldots ,k_{d} \end{array}\right)\prod_{1\leq p\leq d}{x^{\left(p\right)}}^{k_{p}}.$$ (4)

The latent variable of the SVM classifier can then be written as:

$$\begin{array}{ll} \ell & =\sum_{i=1}^{N}\alpha_{i}y_{i}K_{\text{poly}}(x_{i},x)+b\\ & =\sum_{i=1}^{N}\alpha_{i}y_{i}{\left(ax_{i}^{T}x+c\right)}^{\delta }+b=\sum_{i=1}^{N}\alpha_{i}y_{i}{\left(a\sum_{p=1}^{d}x_{i}^{\left(p\right)}x^{\left(p\right)}+c\right)}^{\delta }+b\\ & =\sum_{i=1}^{N}\alpha_{i}y_{i}[c^{\delta }+a^{\delta }\sum_{p=1}^{d}x_{i}^{\left(p\right)}{}^{\delta }x^{\left(p\right)}{}^{\delta }+\sum_{p=1}^{d}\sum_{k_{p}+k_{c}=\delta }\left(\begin{array}{c} \delta \\ k_{p},k_{c} \end{array}\right)a^{k_{p}}x_{i}^{\left(p\right)}{}^{k_{p}}x^{\left(p\right)}{}^{k_{p}}c^{k_{c}}+\\ & \;\sum_{p=1}^{d}\sum_{q\neq p}\sum_{\begin{array}{c} k_{p}+k_{q}=\delta \\ k_{p},k_{q}\neq \delta \end{array}}\left(\begin{array}{c} \delta \\ k_{p},k_{q} \end{array}\right){\left(ax_{i}^{\left(p\right)}x^{\left(p\right)}\right)}^{k_{p}}{\left(ax_{i}^{\left(q\right)}x^{\left(q\right)}\right)}^{k_{q}}+\\ & \;\sum_{p=1}^{d}\sum_{q\neq p}\sum_{\begin{array}{c} k_{p}+k_{q}+k_{c}=\delta \\ k_{p},k_{q},k_{c}\neq \delta \\ k_{p}+k_{q}\neq \delta \\ k_{p}+k_{c}\neq \delta \\ k_{q}+k_{c}\neq \delta \end{array}}\left(\begin{array}{c} \delta \\ k_{p},k_{q},k_{c} \end{array}\right){\left(ax_{i}^{\left(p\right)}x^{\left(p\right)}\right)}^{k_{p}}{\left(ax_{i}^{\left(q\right)}x^{\left(q\right)}\right)}^{k_{q}}c^{k_{c}}+\Delta ]\\ & \;+b\\ & =\sum_{i=1}^{N}\alpha_{i}y_{i}\left(b^{\prime }+\sum_{p=1}^{d}g^{\left(p\right)}\left(x_{i}^{\left(p\right)},x^{\left(p\right)}\right)+\sum_{p=1}^{d}\sum_{q\neq p}g^{\left(p,q\right)}\left(x_{i}^{\left(p,q\right)},x^{\left(p,q\right)}\right)+\Delta \right)+b\;\\ & =\sum_{p=1}^{d}f^{\left(p\right)}\left(x_{i}^{\left(p\right)},x^{\left(p\right)}\right)+\sum_{p=1}^{d}\sum_{q\neq p}f^{\left(p,q\right)}\left(x_{i}^{\left(p,q\right)},x^{\left(p,q\right)}\right)+b^{\prime \prime }+\Delta \ell \end{array}$$

Here, we define *f*(*p*) as the functional form of the *p*^th^ input *x*^(*p*)^, i.e. the contribution to the latent variable that is solely attributed to *x*^(*p*)^. In analogy, *f*^(*p*,*q*)^ is defined as the contribution to the latent variable that is attributed to the combination of inputs *x*^(*p*)^ and *x*^(*q*)^. The derivation above, shows that for each *a*, *c* and *δ*, an SVM classifier with a polynomial kernel can be expanded in main contributions *f*^(*p*)^, contributions *f*^(*p*,*q*)^ involving two input variables and a rest term Δ*ℓ*, including all contributions involving a combination of more than two input variables. From the equations, it can be seen that whenever *d* or *δ* are not higher than 2, the expansion of the polynomial kernel is exact, i.e. Δ*ℓ* = 0. S2 Text indicates how the terms *f*^(*p*)^ and *f*^(*p*,*q*)^ for this polynomial kernel can be calculated.

When using the popular Radial Basis Function (RBF) kernel, the extension is based on a similar approach. The RBF kernel is defined as

$$K_{\text{RBF}}\left(x,z\right)=\exp\left(-\frac{1}{\sigma^{2}}||x-{z||}_{2}^{2}\right)=\exp\left(-\gamma ||x-{z||}_{2}^{2}\right)\;,$$

with $\sigma^{2}=\frac{1}{\gamma }$ the kernel width. Using the Taylor expansion of the exponential function, this kernel can be written as

$$K_{\text{RBF}}\left(x,z\right)=\sum_{n=0}^{\infty }\frac{{\left(-1\right)}^{n}\gamma^{n}(||x-{z||}_{2}^{2}{)}^{n}}{n!}\;.$$

Application of the multinomial theorem results in

$$K_{\text{RBF}}\left(x,z\right)=\sum_{n=0}^{\infty }\frac{{\left(-1\right)}^{n}\gamma^{n}}{n!}\sum_{k_{1}+\cdots +k_{d}=n}\left(\begin{array}{c} n\\ k_{1},\ldots ,k_{d} \end{array}\right)\prod_{1\leq p\leq d}{\left({\left(x^{\left(p\right)}-z^{\left(p\right)}\right)}^{2}\right)}^{k_{p}}\;.$$ (5)

The question whether SVM classifiers using the RBF kernel can be visualized and explained as in Figs 1 and 2 is now reduced to the question whether we can write Eq (5) as the addition of terms only depending on one input variable, or by extension also including terms depending on two input variables. To achieve this, Eq (5) is written as:

$$K_{\text{RBF}}\left(x,z\right)=\sum_{n=0}^{\infty }\frac{{\left(-1\right)}^{n}\gamma^{n}}{n!}\left[\sum_{p=1}^{d}{\left(x^{\left(p\right)}-z^{\left(p\right)}\right)}^{2n}\right.+\left.\sum_{p=1}^{d}\sum_{q\neq p}\sum_{\begin{array}{c} k_{p}+k_{q}=n\\ k_{p},k_{q}\neq n \end{array}}\left(\begin{array}{c} n\\ k_{p},k_{q} \end{array}\right){\left(x^{\left(p\right)}-z^{\left(p\right)}\right)}^{2k_{p}}{\left(x^{\left(q\right)}-z^{\left(q\right)}\right)}^{2k_{q}}\right]+\Delta \;,$$ (6)

$$=\sum_{p=1}^{d}g^{\left(p\right)}\left(x^{\left(p\right)},z^{\left(p\right)}\right)+\sum_{p=1}^{d}\sum_{q\neq p}g^{\left(p,q\right)}\left(x^{\left(p,q\right)},z^{\left(p,q\right)}\right)+\Delta \;.$$ (7)

The latent variable can then be written as:

$$\ell =\sum_{i=1}^{N}\alpha_{i}y_{i}K_{\text{RBF}}\left(x_{i},x\right)+b$$ (8)

$$=\sum_{i=1}^{N}\alpha_{i}y_{i}\left[\sum_{p=1}^{d}g^{\left(p\right)}\left(x_{i}^{\left(p\right)},x^{\left(p\right)}\right)+\sum_{p=1}^{d}\sum_{q\neq p}g^{\left(p,q\right)}\left(x_{i}^{\left(p,q\right)},x^{\left(p,q\right)}\right)+\Delta \right]+b$$ (9)

$$=\sum_{p=1}^{d}f^{\left(p\right)}\left(x_{i}^{\left(p\right)},x^{\left(p\right)}\right)+\sum_{p=1}^{d}\sum_{q\neq p}f^{\left(p,q\right)}\left(x_{i}^{\left(p,q\right)},x^{\left(p,q\right)}\right)+b^{\prime \prime }+\Delta \ell \;.$$ (10)

The kernel function can thus be written as a part dependent on single inputs, i.e. the first term in the above equation, a part depending on two inputs, i.e. the second term, and a rest part, including terms depending on a combination of more than two inputs. A formal definition of these terms is given in S2 Text. The only question that remains is whether the rest term is small enough to be ignored without preformance reduction. The experiments in the next Section illustrate whether and when this is the case. Whenever Δ*ℓ* can be ignored, the SVM model can be visualized by use of color based nomograms, representing *f*^(*p*)^ by color bars, *f*^(*p*,*q*)^ by color plots and converting the latent variable (visualized via the score) into a risk estimate by means of another color bar. A more detailed explanation of how the color based nomogram is constructed from the above expansion is presented in S1 Text.

## Results

In this Section, it is investigated whether it is possible to approximate the SVM model by an additive model, only including terms that can be visualized. Stated otherwise, the SVM model will be approximated using the expansions obtained in the previous sections and ignoring the rest term Δ*ℓ*. This approach is illustrated on several simulated and real-life datasets. Since the proposed visualization method is exact for two-dimensional problems, only problems with at least three input variables will be discussed here. The first examples are based on artificial datasets with only 3 input variables, of which only two are relevant. Each artificial dataset contains 1000 observations, 500 of which are used for training, the remainder for testing the SVM model. It is assumed that, since only two inputs are necessary for the true classification, the approximation method should be able to explain the resulting SVM models: if one input is irrelevant, then why would a well performing SVM model use contributions that involve this input variable? The artificial problem settings are illustrated in S3 Text, where the class separation is illustrated in the two relevant dimensions. We conclude with an application on three real-life datasets from the UCI depository [27] (Fisher Iris, Pima Indians diabetes and German credit risk data).

When using SVMs in combination with an RBF or polynomial kernel, multiple parameters need to be set: (i) the regularization constant *C*, (ii) the width $\frac{1}{\gamma }$ of the RBF kernel or the degree *δ* of the polynomial kernel. For simplicity, we keep both the scale *a* and the bias *c* of the polynomial kernel equal to 1. A grid search in combination with 10-fold cross validation is used to select the optimal parameter set on the training set.

In all the examples we use a fixed grid to tune the parameters. The regularization constant *C* is varied over [10^−7^, 10^2^], using ten steps (exponential grid). The inverse kernel width *γ* is varied over [2^−7^, 2^2^], using ten steps (exponential grid). The degree of the polynomial kernel can range from 1 to 4. Class probabilities are obtained by means of a sigmoid function, the parameters of which are fitted using 3-fold cross-validation on the training data (the default in the R package kernlab). Parameter tuning is performed using the R package caret [33]. The R package kernlab [34] is used to train the SVMs.

Regarding the visualizations, it is opted to show the plots with contributions that are shifted such that the median of all contributions is zero. A diverging colormap is used such that a value of zero corresponds to a white color, negative values are represented in blue and positive values in red. The most important input variables are those with the largest color range.

**Fig 3:**
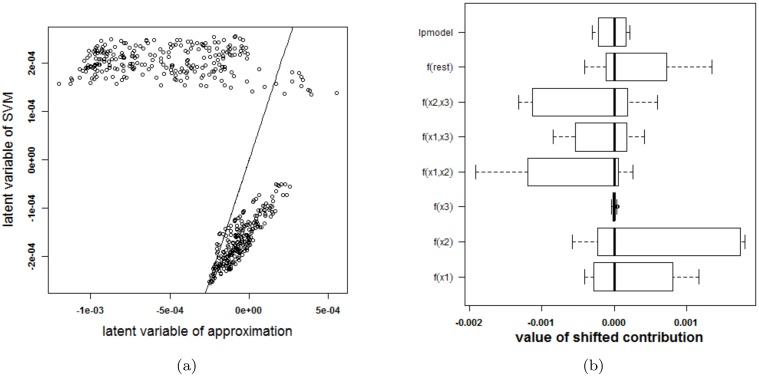
Performance of the approximation method (i.e. the expansion without the rest term Δ*ℓ*) on the two circles data. (a) Latent variables of the SVM model with RBF kernel and the approximation. The approximation is not able to approximate the latent variables of the SVM model. (b) Contributions of the approximation of the SVM model and the rest term. The box-plots visualize the range of the different contributions. The upper boxplot indicates the range of the latent variable of the SVM model. In this example the range of the rest term cannot be ignored in comparison with the ranges of the other contributions. As such, the approximation of this specific SVM model cannot serve as an explanation of the SVM model.

Before continuing to the experiments, it is stressed that visualizing an SVM model based on the proposed approximation and extracting how the SVM model works based on this approximation is only possible after confirmation that the approximation is valid: the rest term should be small. Otherwise, conclusions drawn from the approximation cannot be assumed to be correct. In order to check the validity of the approximation, two types of plots are provided. A first plot relates the latent variable of the SVM model with the approximated latent variable (i.e. the latent variable of the SVM model without the rest term Δ*ℓ*). An example of this type of plot is given in Fig 3(a). The straight line in the plot indicates where the points should be located when the approximation is valid. A second plot represents the ranges of all contributions in the expansion of the latent variable. The range of the latent variable of the SVM model (indicated by lpmodel) is given as well. Whenever the approximation is valid, the rest term will be small in comparison with the latent variable. An example of this plot is given in Fig 3(b).

### Artificial examples

#### Two circles problem

Consider a dataset with three input variables, only two of which are relevant. The observations are all elements of one of two classes. All elements of one class are located on a circle and both circles are concentric. See S3 Text for a visualization of this setting. The SVM classifier with the highest cross-validation performance using an RBF kernel has parameter values *γ* = 2 and *C* = 10^−5^. The resulting classifier is able to classify the training samples perfectly, i.e. an accuracy of 100%. The same accuracy is obtained for the test set.

To check whether the proposed method is able to explain the SVM classifier, the latent variables of the SVM model and the approximation are plotted against each other in Fig 3(a). Only 41% of the training datapoints get the same class label by the approximation and the SVM model. To explain this malfunctioning, the individual terms in the approximation and the rest term are reported in Fig 3(b). The range of the latent variable of the SVM model (indicated as lpmodel) is added as a reference. Since the range of the rest term is large in comparison with the other terms, the rest term can not be ignored. As a result, the approximation method proposed here will not be able to explain the SVM model.

This experiment raises the question how the SVM achieves a good performance despite taking non-relevant information (i.e. higher-order terms including input variables of which it is known that they are irrelevant) into account. To answer this question, the correlation between the rest term and the different terms in the approximation are studied (results not shown). The rest term is highly correlated with the interaction between *x*^(1)^ and *x*^(2)^ (Pearson correlation coefficient = -0.993). The rest term can thus be explained as an interaction between the first two input variables and not as a higher order interaction effect as was expected. As such, dropping the rest term in the approximation results in loss of information.

**Fig 4:**
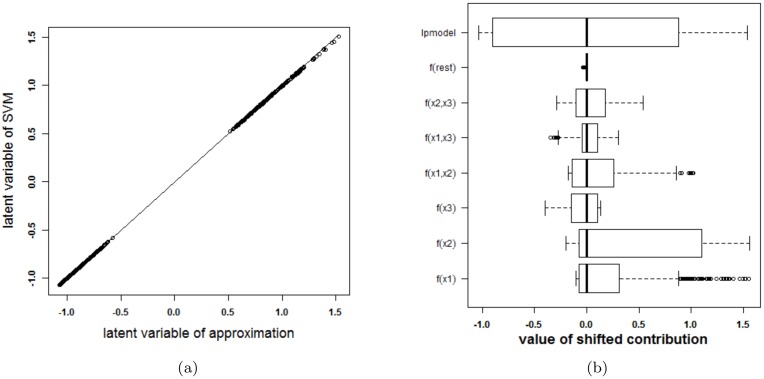
Performance of the approximation method (i.e. the expansion without the rest term Δ*ℓ*) on the two circles data (second SVM model). (a) Latent variables of the SVM model with RBF kernel and the approximation. The approximated latent variable is a good estimate of the latent variable of the SVM model. (b) Contributions of the approximation of the second SVM model and the rest term. The box-plots visualize the range of the different contributions. The upper boxplot indicates the range of the latent variable of the SVM model. In this example the range of the rest term can be ignored in comparison with the ranges of the other contributions. As such, the approximation of this specific SVM model will be able to explain the classifier.

**Fig 5:**
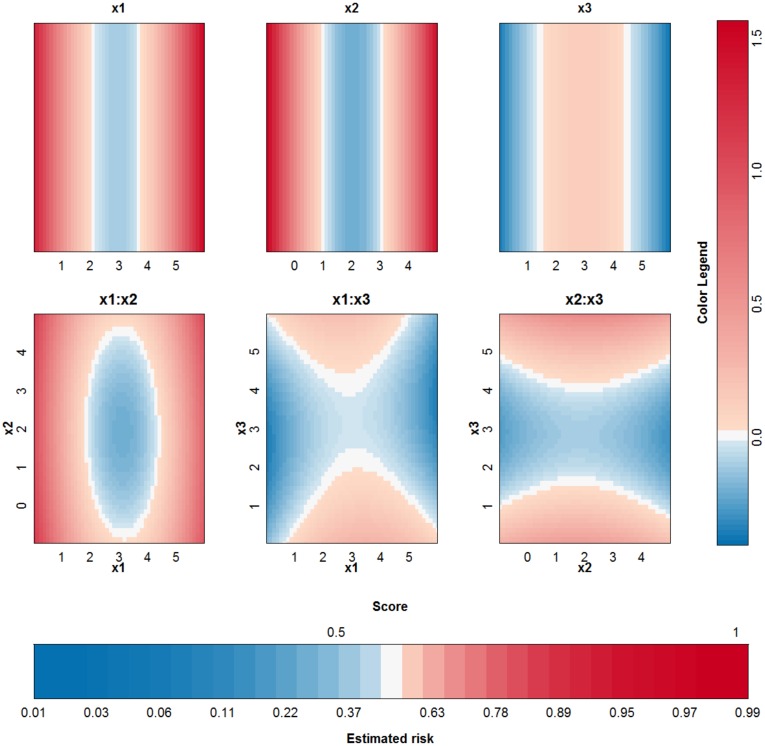
Visualization of the second SVM model with RBF kernel on the example of the two circles.

To investigate whether the above problem is due to the SVM model or due to the expansion in individual terms, other SVM models, with other tuning parameters but similar cross-validation results, are analysed. The same 10-fold cross validation performance can be achieved from a variety of parameter values. As such, the approximation method is applied a second time. This time, a cross-validation performance of at least 95% of the optimal performance is required and the kernel width should be as large a possible. The validity of the approximation of this second SVM model, with parameter values *γ* = 2^−7^ and *C* = 10, is analysed in Fig 4. The approximation is very good and the rest term can be ignored. As such, the proposed approximation is able to explain the SVM classifier. The visualization of this model (see Fig 5) reveals that the contributions of *x*^(1)^, *x*^(2)^ and their interaction are most important. This SVM classifier and the approximation obtain a performance accuracy of 100% on training and test set.

**Fig 6:**
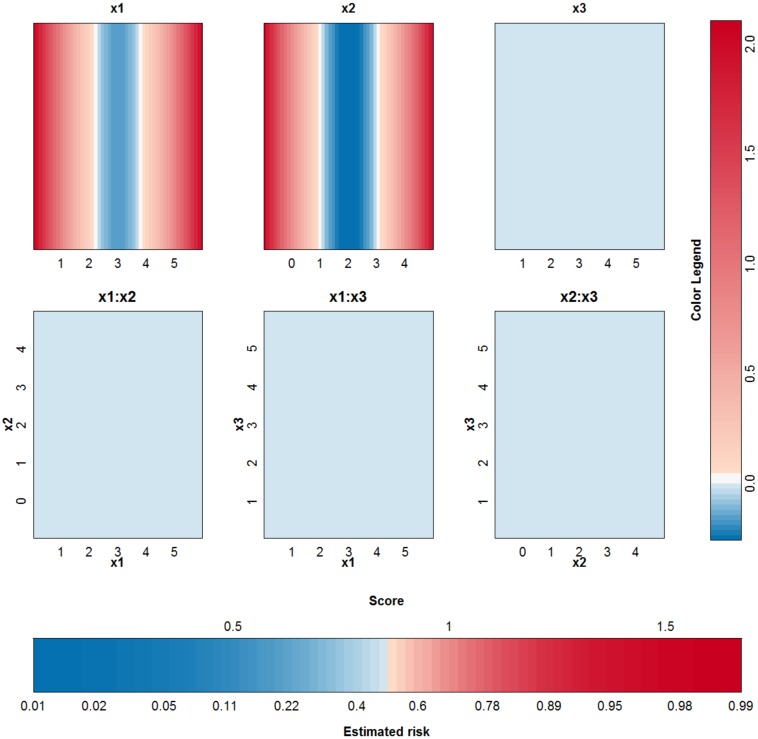
Visualization of the third SVM model (polynomial kernel) on the example of the two circles.

To compare the working of different kernels, the polynomial kernel is used on the same example. The best performing kernel had a degree *δ* = 2 and the regularization constant was *C* = 0.01. The approximation is able to perfectly explain the SVM model since the degree of the kernel is not larger than 2. The SVM model is approximated by the terms visualized in Fig 6. Comparison with Fig 5 instantly shows that a polynomial kernel is better suited for the job: the value of the irrelevant input *x*^(3)^ is not used by the polynomial kernel. Only the main effects of the first two input variables are necessary to obtain a classifier with 100% accuracy.

**Fig 7:**
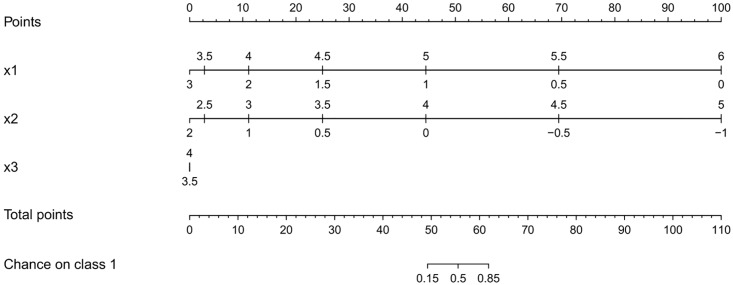
Nomogram of a logistic regression model including polynomial transformations of the input variables for the two circles problem. The non-linearities are visualized by the use of two axes for each input.

To compare the proposed color based nomogram with existing tools for logistic regression, a logistic regression model allowing polynomial transformations of the input variables is trained using the lrm function within the rms package in R [30]. The model includes polynomial transformations of all input variables of the first and second degree. The resulting nomogram is represented in Fig 7. The model results in an accuracy of 100% on training and test set. The non-linear relationship is visualized by repetition of the input axis.

#### Swiss roll problem

As a second example, an SVM model is trained on the artificial two-class swiss roll example. The data are points in a three-dimensional space and non-linearly separable in a 2-dimensional plane spanned by *x*^(1)^ and *x*^(2)^. The third variable is again irrelevant. See S3 Text for an illustration of this setting.

**Fig 8:**
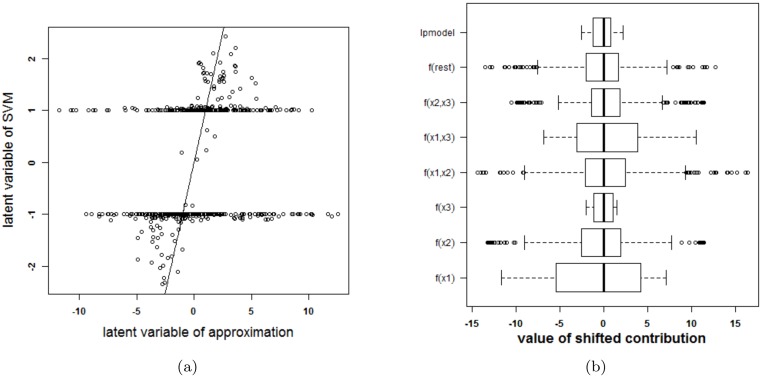
Performance of the approximation method (i.e. the expansion without the rest term Δ*ℓ*) on the swiss roll problem. (a) Latent variables of the SVM model with RBF kernel and the approximation. The approximation is not able to approximate the latent variables of the SVM model. (b) Contributions of the approximation of the SVM model and the rest term. The box-plots visualize the range of the different contributions. The upper boxplot indicates the range of the latent variable of the SVM model. In this example the range of the rest term cannot be ignored in comparison with the ranges of the other contributions. As such, the approximation of this specific SVM model cannot serve as an explanation of the SVM model.

The tuning parameters resulting in the largest 10-fold CV performance for the SVM model with RBF kernel are *γ* = 4 and *C* = 10. The latent variable of the SVM model and the approximation as well as the ranges of the contributions, the rest term and the latent variable of the SVM model are shown in Fig 8. One can clearly see that the approximation is not able to explain the SVM classifier. The rest term cannot be ignored in this case.

In contrast with the previous artificial example, looking for another optimal parameter pair does not yield a satisfying solution. This can be explained by the fact that the swiss roll example is highly non-linear and a small kernel width is necessary to obtain a good performance. To have a small rest term, a large kernel width is necessary, to reduce the influence of higher-order interaction terms. Scatterplots between all contributions and the rest term (plot not shown) explain why dropping the rest term has such a dramatical effect: the rest term is highly (inversely) correlated with the contribution of the interaction between *x*^(1)^ and *x*^(2)^ (Pearson correlation coefficient = -0.968). Investigation of the Pearson correlations between the terms of the approximation reveals that the contribution of the interaction between *x*^(1)^ and *x*^(3)^ is highly (inversely) correlated with the contribution of *x*^(1)^ (Pearson correlation coefficient = -0.965) and the contribution of the interaction between *x*^(2)^ and *x*^(3)^ is highly (inversely) correlated with the contribution of *x*^(2)^ (Pearson correlation coefficient = -0.962). The rest term in this example is seemingly used to counter-balance other contributions. The same is true for the interaction terms involving *x*^(3)^. All these findings indicate that the third input variable might be dropped before building the SVM. Results of this approach are not shown here since this yields a two-dimensional problem for which the approximation is exact.

Note that for the example on the two circles, selection of the most relevant input variables is an alternative (and probably preferred) to the solution proposed in the previous Section.

#### Checkerboard problem

**Fig 9:**
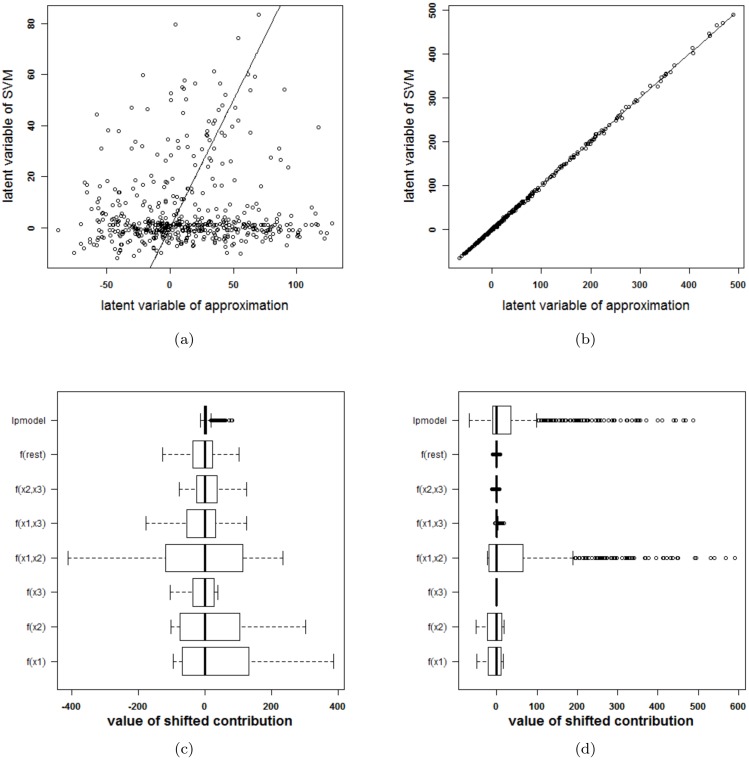
Comparison of the performance of the approximations (i.e. the expansion without the rest term Δ*ℓ*) of two SVM models on the checkerboard problem. (a)-(c): RBF kernel, (b)-(d): polynomial kernel. (a)-(b): Latent variable of the approximation versus latent variable of the original SVM model. (c)-(d): Range of all contributions in the approximation, the rest term and the latent variable of the SVM model. For the RBF kernel, the rest term is much larger than the latent variable, resulting in an approximation that is unable to explain the SVM model. For the polynomial kernel, the rest term is negligible in comparison with the other terms and the approximation is nearly perfect.

**Fig 10:**
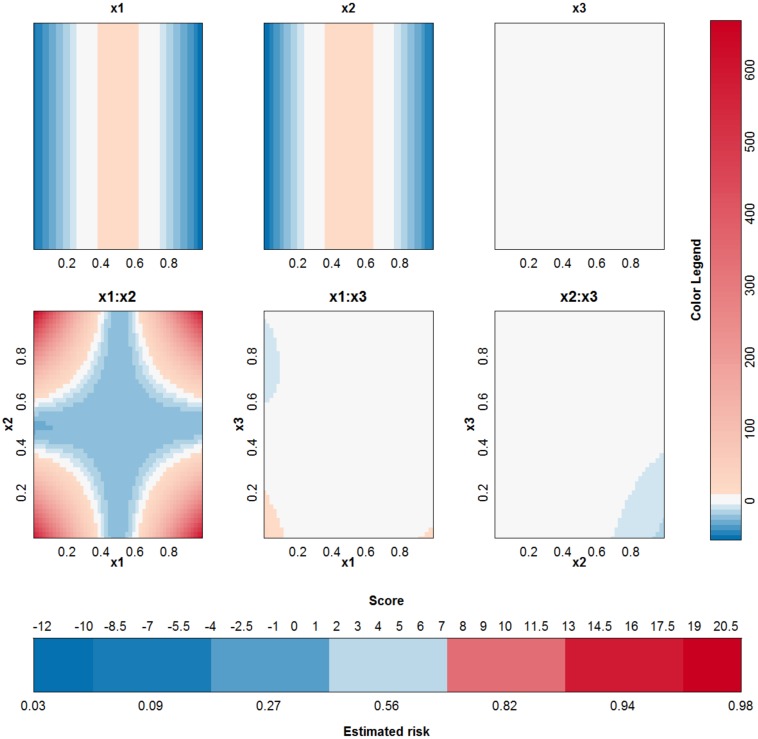
Visualization of the SVM model with polynomial kernel on the checkerboard example. It can be seen that all contributions involving *x*^(3)^ do not contribute in a large extent since the range of these contributions is very small in comparison with the other contributions.

This last artificial example illustrates that in some cases it is possible to explain an SVM model using one kernel type, while it is not possible to explain an SVM model trained on the same data using another kernel type. To illustrate this, a checkerboard problem with 9 blocks in the plane spanned by *x*^(1)^ and *x*^(2)^ is used. A third input variable is again irrelevant. A visualization of this setting is provided in S3 Text. The two SVM models with optimal tuning parameters are: a first SVM model with an RBF kernel (*γ* = 2^−4^ and *C* = 10^5^) and a second SVM model with a polynomial kernel (*δ* = 4 and *C* = 100). For both models, the optimal tuning parameters by means of 10-fold cross validation are used. The latent variables and the terms in the approximations for both models are given in Fig 9. The accuracy of the SVM with RBF kernel on the training data is 0.96% and on the test data 0.91%. The accuracy of the SVM with polynomial kernel on the training data is 0.99% and on the test data 0.96%. The SVM model with an RBF kernel and its approximation agree on the class label in only 56% of the observations in the training data. The use of another parameter pair does not yield a better approximation. For the polynomial kernel, the agreement is 99%. This performance difference of the approximation method is also seen in Fig 9, where a very large rest term is noted for the expansion of the SVM with RBF kernel and a very small rest term is seen when using the polynomial kernel. Fig 10 illustrates the visualization of the SVM model with the polynomial kernel. This visualization is very valuable since it clearly indicates that the third variable is not necessary and the estimated functional forms of the other effects are correct.

#### Overlapping Gaussians

**Fig 11:**
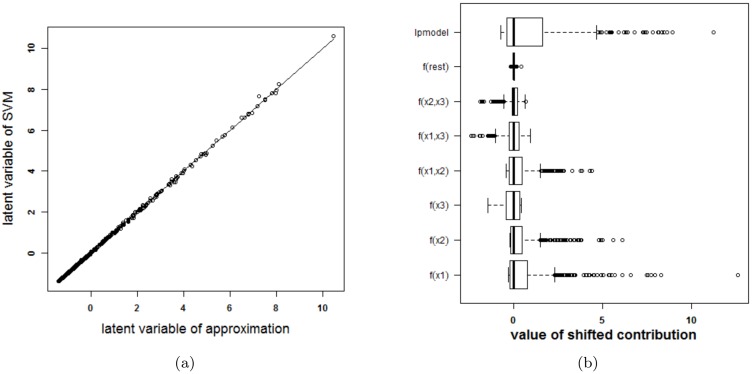
Performance of the approximation method (i.e. the expansion without the rest term Δ*ℓ*) on the two Gaussians data. (a) Latent variables of the SVM model with RBF kernel and the approximation. The approximated latent variable is a good estimate of the latent variable of the SVM model. (b) Contributions of the approximation of the SVM model and the rest term. The box-plots visualize the range of the different contributions. The upper boxplot indicates the range of the latent variable of the SVM model. In this example the range of the rest term can be ignored in comparison with the ranges of the other contributions. As such, the approximation of this specific SVM model will be able to explain the classifier.

**Fig 12:**
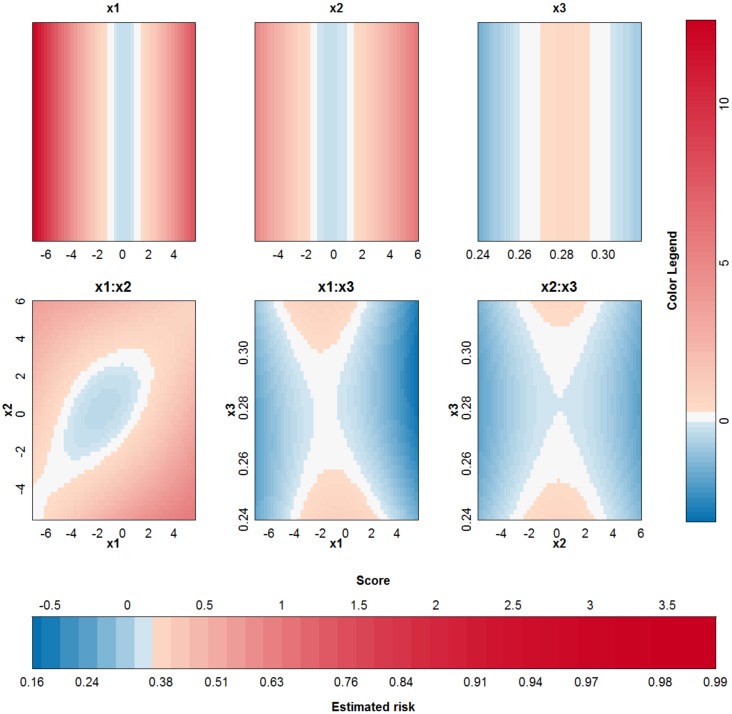
Visualization of the SVM model with RBF kernel on the example of the two Gaussians.

In this last artificial problem a classification problem of two strongly overlapping classes is used. The data of these two overlapping Gaussians are illustrated in S3 Text. For an SVM model using an RBF kernel the optimal tuning parameters by means of 10-fold cross validation are *γ* = 2^−6^ and *C* = 100. The latent variables and the terms in the approximations for both models are given in Fig 11. Since the rest term is very small, the approximation can be used to explain the SVM model. The accuracy of the SVM with RBF kernel on the training data is 78% and on the test data 75%. The accuracy of the approximation on the training data is 78% and on the test data 74%. The SVM and the approximation agree on 100% of the cases in the training set and on 99.8% of the cases in the test set. Fig 12 illustrates the visualization of the SVM model.

### Real life data

#### The IRIS dataset

**Fig 13:**
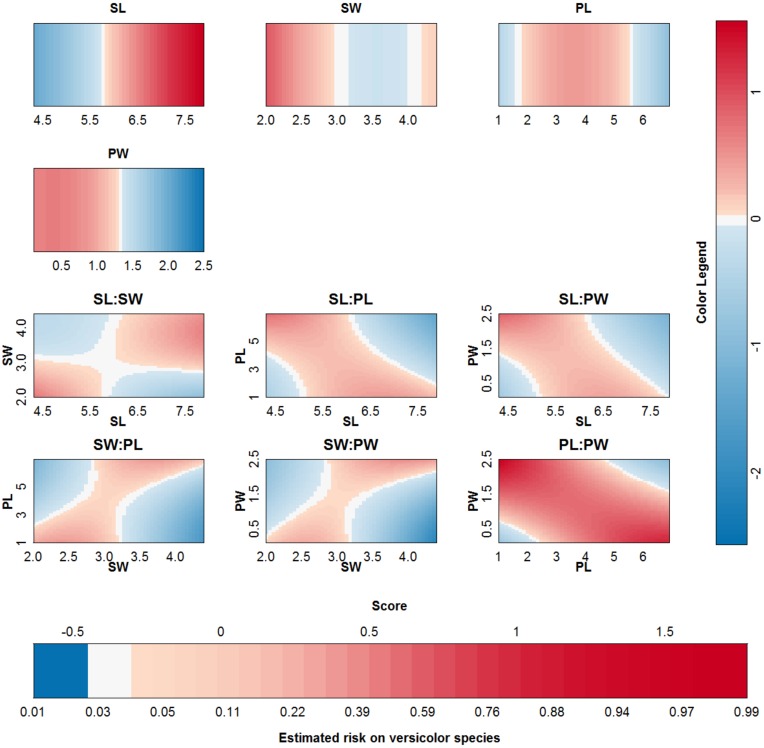
Visualization of the SVM model on the IRIS data set.

**Fig 14:**
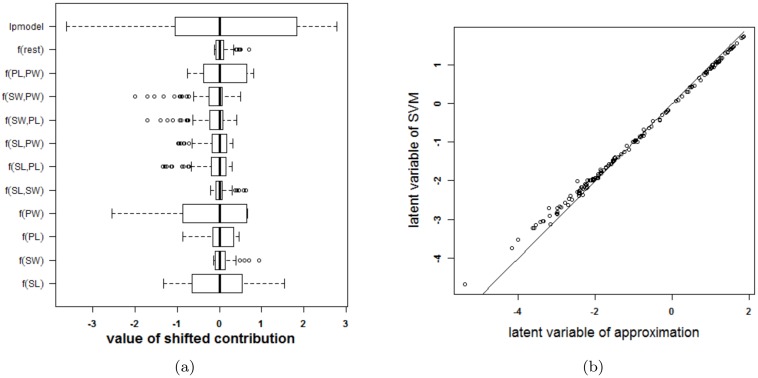
Performance of the approximation method (i.e. the expansion without the rest term Δ*ℓ*) on the IRIS data. (a) Boxplots of the contributions of the approximation of the SVM model, the rest term and the latent variable of the SVM model. The range of the rest term can be ignored in comparison with the ranges of the other contributions. (b) Latent variable of the original model versus those obtained from the approximation. The approximation is able to estimate the latent variable of the SVM model very accurately and as such can be used to explain the SVM model.

The proposed approach is applied to the IRIS dataset [27, 35] and using an RBF kernel. This data contains information on 150 cases, four input variables and a class label. The labels are setosa, versicolor and virginica. For the purpose of this work, the output label was defined as species of type versicolor. An SVM was trained on 100 randomly chosen observations and tested on the remaining 50. The classifier obtained from parameter values that achieve the highest 10-fold cross validation performance (*γ* = 2^−5^ and *C* = 100) is represented in Fig 13. The SVM achieves an accuracy of 99% and 96% on training and test set respectively. The approximation achieves an accuracy of 98% and 96% on training and test set respectively. The validity of the approximation is illustrated in Fig 14. Since the rest term is very small, the approximation yields latent variables that are very close to the latent variables obtained by the original SVM model. The approximation performs very good in this case and agrees on class labels with the SVM model in 98% of the training cases and in 100% of the test cases. From the visualization of the model it is seen that sepal length (SL), petal length (PL) and petal width (PW), and the interaction between PL and PW contribute the most to the latent variable (colors range to the extremes of the color legend). This is also confirmed by investigating the ranges of the contributions (see Section Discussion for a discussion on the importance of the latter.) A bivariate plot (see S1 Fig) indicates that PL and PW are most important for class separation in a linear setting. The interaction between PL and PW is also valuable.

#### The Pima Indians data set

**Fig 15:**
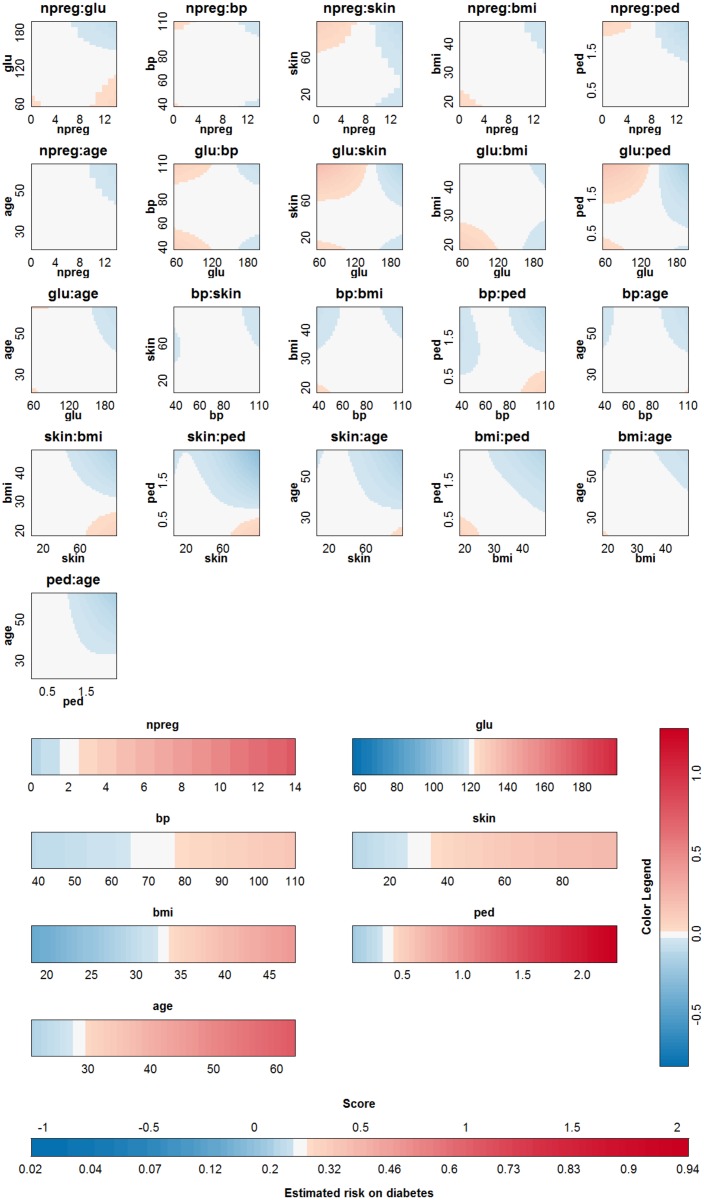
Visualization of the SVM model on the Pima data set.

**Fig 16:**
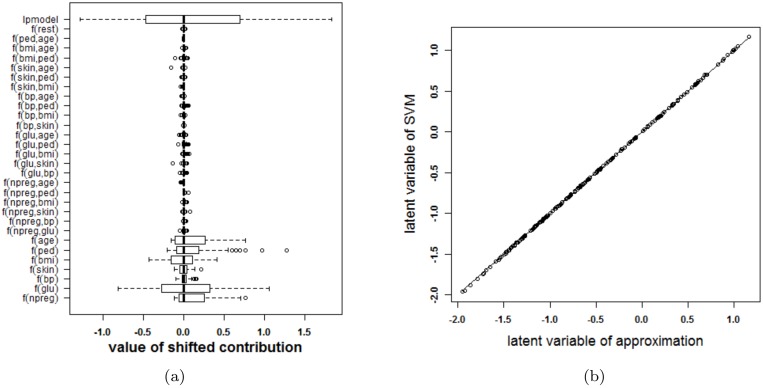
Performance of the approximation method (i.e. the expansion without the rest term Δ*ℓ*) on the Pima data. (a) Boxplots of the contributions of the approximation of the SVM model, the rest term and the latent variable of the SVM model. The range of the rest term can be ignored in comparison with the ranges of the other contributions. (b) Latent variable of the original model versus those obtained from the approximation. The approximation is able to estimate the latent variable of the SVM model very accurately and as such can be used to explain the SVM model.

The Pima Indians dataset [27, 29] contains 532 cases with complete information for women who were at least 21 years old, of Pima Indian heritage and living near Phoenix, Arizona. Seven different input variables were available: number of pregnancies (npreg), plasma glucose concentration (glu), diastolic blood pressure (bp), triceps skin fold thickness (skin), body mass index (bmi), diabetes pedigree function (ped) and age. The outcome is whether or not these women have diabetes according to World Health Organization criteria. The SVM model with RBF kernel was trained on a random set of 200 of these women, as provided as a training set in the R package MASS [29]. The classifier obtained from parameter values that achieve the highest 10-fold cross validation accuracy (*γ* = 2^−7^ and *C* = 1) is represented in Fig 15. The accuracy of the approximation is illustrated in Fig 16. Since the rest term is very small, the approximation yields latent variables that are very close to the latent variables obtained by the original SVM model. The approximation performs very good in this case and agrees on the estimated class labels with the SVM model in 100% of the cases. The SVM achieves an accuracy of 78% on training and test set. The approximation also achieves an accuracy of 78% on training and test set. From the visualization of the model it is seen that the interaction effects are of minor importance (very light colors for all ranges of the input variables). The main effects of blood pressure and skin thickness are less important than the other main effects. Comparing this result with feature selection methods in the literature confirms these results. In [21] 12 feature selection methods from the literature are compared on the Pima dataset. The blood pressure and the skin thickness are selected three times among these 12 methods, whereas all other variables are selected at least four times. In [36] different feature selection methods are also compared on the Pima dataset. Ranking features according to their importance (see Table 3 in [36]) also indicates that the blood pressure and the skin thickness are least important.)

#### German credit risk data

**Fig 17:**
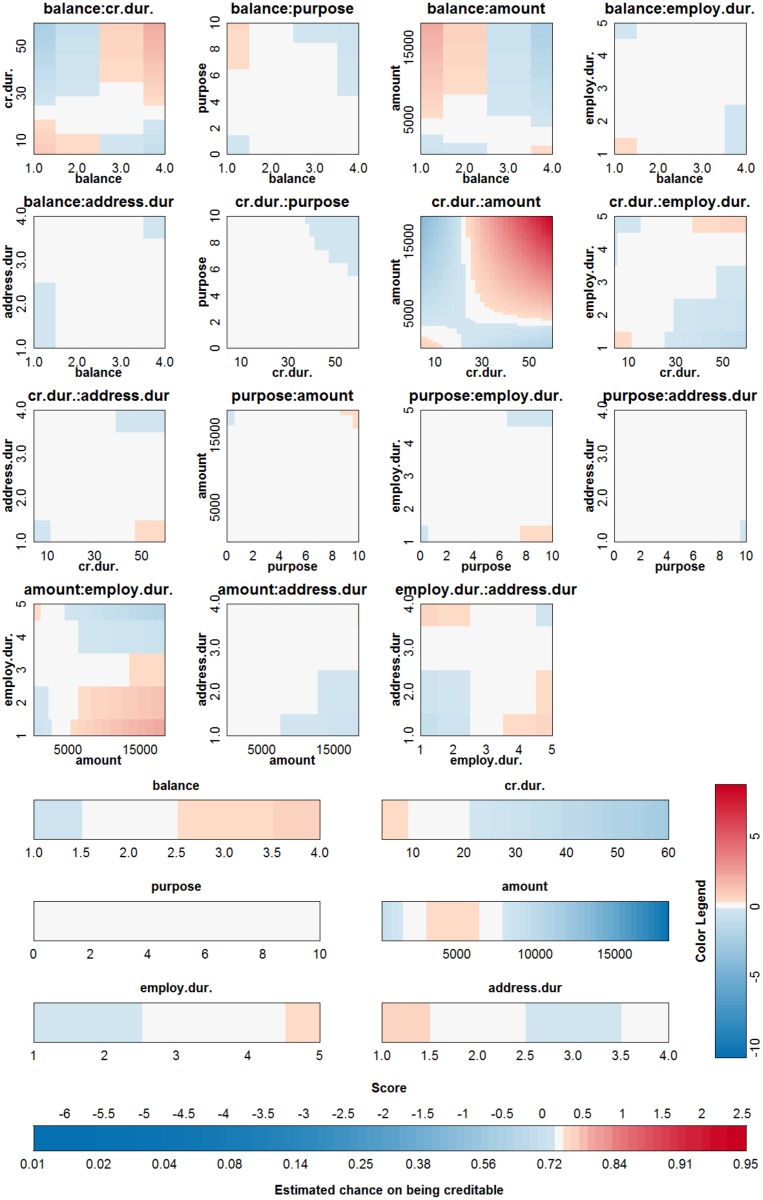
Visualization of the SVM model with polynomial kernel on the German credit risk data set.

**Fig 18:**
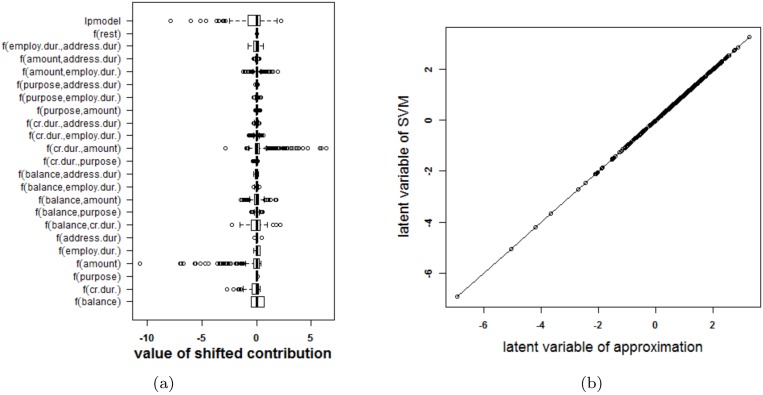
Performance of the approximation method (i.e. the expansion without the rest term Δ*ℓ*) on the German credit risk data. (a) Boxplots of the contributions of the approximation of the SVM model, the rest term and the latent variable of the SVM model. The range of the rest term can be ignored in comparison with the ranges of the other contributions. (b) Latent variable of the original model versus those obtained from the approximation. The approximation is able to estimate the latent variable of the SVM model very accurately and as such can be used to explain the SVM model.

**Fig 19:**
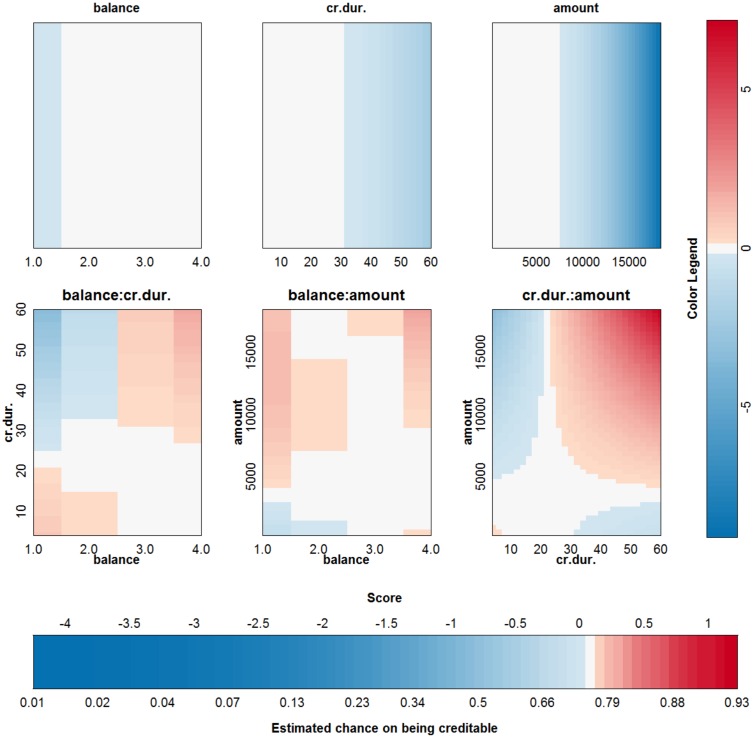
Visualization of the second SVM model on the German credit risk data set.

**Fig 20:**
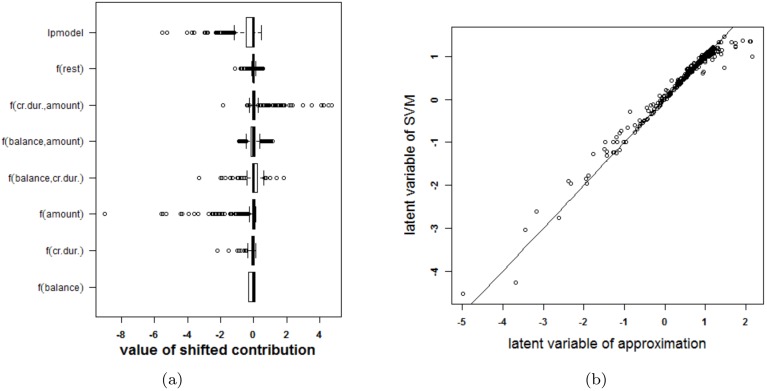
Performance of the approximation method (i.e. the expansion without the rest term Δ*ℓ*) on the German credit risk data (using only three inputs). (a) Boxplots of the contributions of the approximation of the SVM model, the rest term and the latent variable of the SVM model. The range of the rest term can be ignored in comparison with the ranges of the other contributions. (b) Latent variable of the original model versus those obtained from the approximation. The approximation is able to estimate the latent variable of the SVM model accurately and as such can be used to explain the SVM model.

An SVM with a polynomial kernel is used to illustrate the approach on the German credit risk data [27]. The data is taken from https://onlinecourses.science.psu.edu/stat857/node/215, and a random subset of 500 observations is used to train the SVM, the remaining 500 observations are used to test the SVM. In this example only 6 inputs are selected to predict the creditability of the applicants: the status of applicant’s account in the bank (balance, categorical input), the duration of the credit in months (cr.dur.), the purpose of the credit (purpose), the amount of credit asked for (amount), the duration of the applicant’s present employment (employ.dur.), and the applicant’s duration of residence (address.dur.). The classifier obtained from parameter values that achieve the highest 10-fold cross validation accuracy (*δ* = 2 and *C* = 1) is represented in Fig 17. Since the degree of the polynomial kernel equals 2, the visualization is an exact representation of the SVM model. The accuracy on the training and test set are 75% and 76% respectively. Looking at the interaction effects, it is clear that most of the interactions are not relevant (white color in the graph). Taking the range of the contributions into account (see Fig 18) illustrates that the most important effects are the effects of balance, credit duration, amount of the credit and interactions between these. A second SVM is built using only these 3 inputs, resulting in a polynomial kernel of the third degree and a regularization constant *C* = 0.01. The model is visualized in Fig 19 and the correctness of the representation is illustrated in Fig 20. It is clear that the approximation yields latent variables that are in line with those obtained from the SVM classifier. The accuracy of the model and approximation on the training set is 73%, for the test set this accuracy is 76%. The approximation and the SVM model agree on the predicted class label in 99% of the cases.

**Fig 21:**
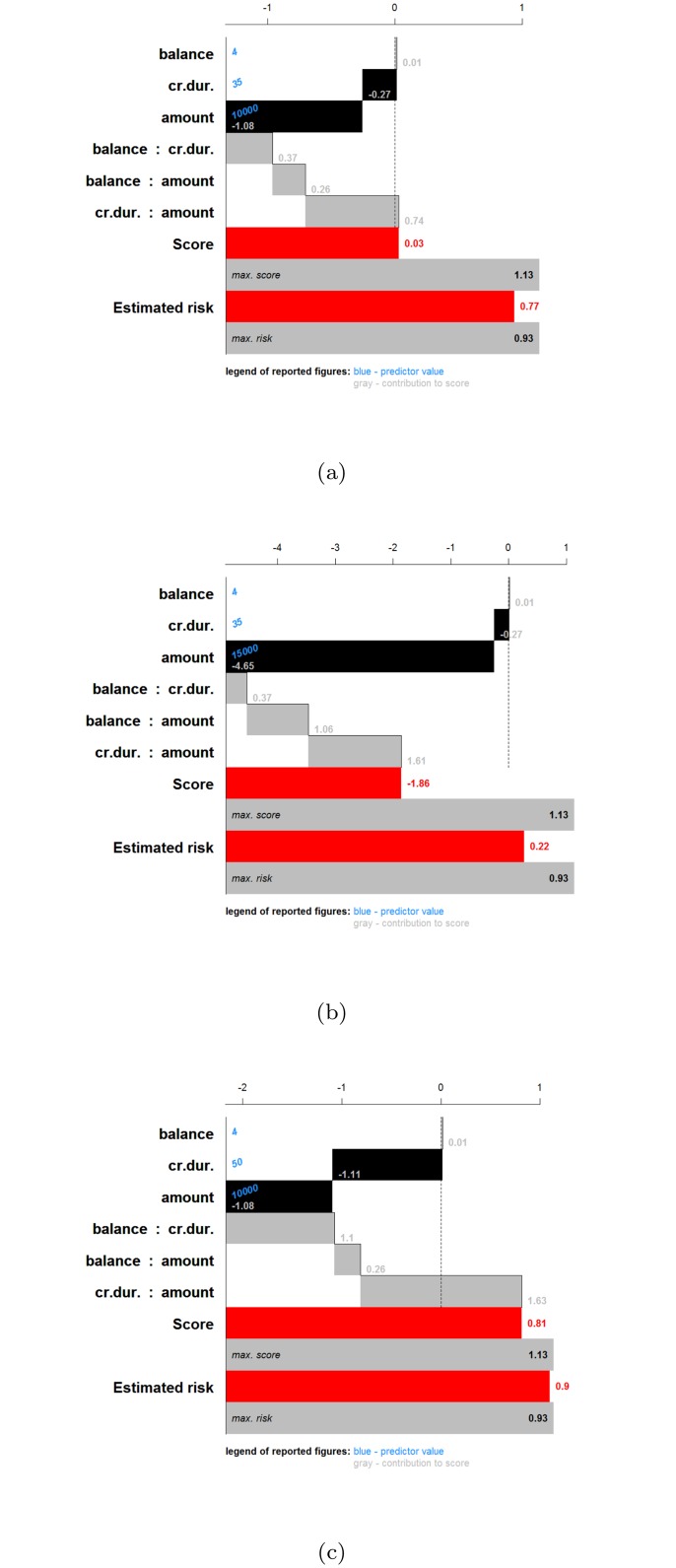
Cumulative contribution charts for three applicants to illustrate the effect of the SVM model on the German credit risk data. The bars indicate the value of the contributions. (a) applicant 1 (balance = 4, credit duration = 35, credit amount = 10000), (b) applicant 2 (balance = 4, credit duration = 35, credit amount = 15000), (c) applicant 3 (balance = 4, credit duration = 50, credit amount = 10000).

What can we learn from this representation? It has to be noted that all effects containing the same input should be considered together when interpreting the results. With more than 2 inputs, this is however impossible, so the effects should be interpreted considering all other inputs constant. For interaction effects the interpretation is harder since one input can occur in more than one interaction effect. For this example it is noted that having a higher balance on the current account increases the chance of being creditable. A longer duration of the credit decreases this chance. The same effect is noted for an increasing credit amount. Looking at the interaction effect of balance and credit duration, it is noted that for a duration larger than 20–25 months, the main effect of the balance on the account is amplified, whereas for a short credit duration the effect of balance is opposite to the main effect. For a low balance on the current account, an increasing credit amount increases the chance of being creditable. This seems counterintuitive, however this increase (increase in points indicated by the color legend) is lower than the decrease indicated in the main effect of the credit amount. As such, the interaction is making a small correction w.r.t. the main effect, depending on the balance. From the interaction effect between credit amount and credit duration it could be concluded that a higher credit duration and amount increases the chance of being creditable. However, this interpretation leaves out the main effects of the involved inputs. To aid in this complex interpretation, the contributions of all effects are plotted (Fig 21) for three applicants: applicant 1 (balance = 4, credit duration = 35, credit amount = 10000), applicant 2 (balance = 4, credit duration = 35, credit amount = 15000), applicant 3 (balance = 4, credit duration = 50, credit amount = 10000). This type of summary for one observation was proposed in [23] and is related to the work in [37, 38]. The displayed charts illustrate how the chances on creditability are obtained from the different input values. Comparison of the charts for different applicants illustrates the change in effects of the inputs. Comparing applicant 1 and 2 reveals that the increase in amount implies a decrease in the contribution of amount by 3.57 points, the effect of the interaction between balance and amount increases by 0.8 points and the effect of the interaction between amount and credit duration increases by 0.87 points. The result is a reduction of the creditability of the applicant. Comparing applicant 1 and 3 indicates that an increase in the credit duration yields a reduction of 0.84 points for the main effect of credit duration, an increase of 0.73 points due to the interaction of credit duration and balance, and an increase of 0.89 points due to the interaction of credit duration and amount. As such, an increase of the credit duration would increase the chance of being creditable.

**Fig 22:**
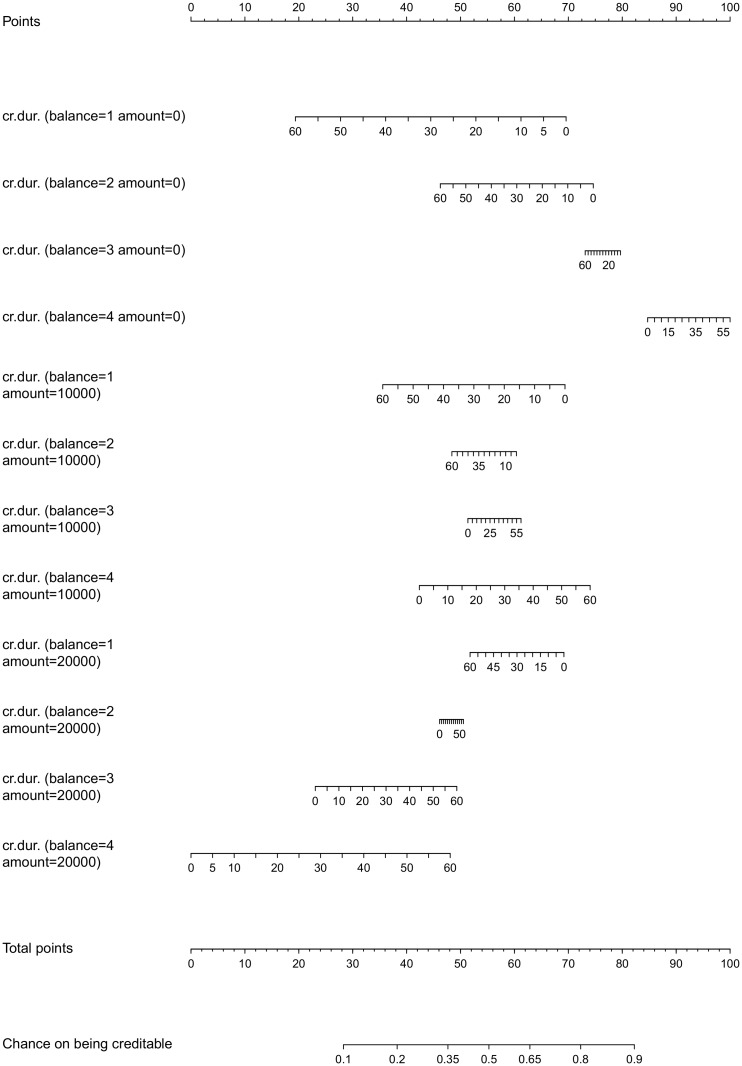
Nomogram of a logistic regression model including linear main and interaction effects for the German credit risk problem with a reduced input set and 3 categories for the amount. Interactions are dealt with by grouping main and interaction effects containing the same inputs. Interactions between continuous inputs are only possible after categorization of at least one of these inputs.

To compare the proposed color based nomogram with the standard nomogram when dealing with non-linearities and interactions, a nomogram was created for a logistic regression model including only linear main and interaction effects for the reduced set of input variables. The lrm function within the rms package in R [30] was used for this purpose. The resulting nomogram is represented in Fig 22. To create this nomogram, it was necessary to categorize continuous input variables (i.e cr.dur. and amount). To make the graph readable, only 3 categories for amount were used. For all inputs involved in an interaction, combinations of input variables are made such that for each input only one point needs to be read from the graph. Since all inputs interact with each other in this example, only one point needs to be read from the graph. An obvious disadvantage of this representation, is the loss of information due to the categorization of continuous inputs. Additionally, it was impossible to represent a full model in a one page graph, since the number of possible combinations becomes too large. Getting a global view on the risk prediction process from this representation is less straightforward as with the presented color based technique. The accuracy of this model on training and test set is 72% and 77% respectively.

In [39] a feature selection algorithm indicated that out of the 20 available features, status, credit duration, credit history, credit amount, savings, housing and foreign worker, were the 7 selectable features. From the selected set of features that we used, only credit duration and credit amount also occur in this set. This agrees with the selection obtained after interpreting the visualization of the SVM model, indicating balance, credit duration and credit amount as most important features.

## Software

All functionality to perform the analyses from this manuscript is provided as an R package https://cran.r-project.org/web/packages/VRPM/. The package also includes two applications that enable to play with the methods for the IRIS and Pima datasets, such that the interested user can have a look at the possibilities of the method before learning to use the package. The software provides additional functionalities. Firstly, the color map can be chosen: a rainbow color map, a sequential color map (a single color with changing intensity), a diverging color map (with two different colors and white in the middle, as used throughout this work), a black-and-white color map, or the viridis color map. Secondly, the level of the contributions that is represented as zero can be set to zero, mean, median (as in this work), or minimum (as is done in a classical nomogram). Thirdly, the range of the input variables can be chosen to reduce the effect of outliers on the visualization of the approximation and as such on the interpretation (see the discussion). Fourthly, one specific observation can be added to the representation to visualize how the risk prediction for this observation is built up using the approximation of the SVM model. A movie illustrating all these functionalities is provided in S1 Video.

## Discussion

This manuscript provides a way to explain how support vector machines using RBF and polynomial kernels generate decisions for unseen data. This approach is completely new and raises a lot of questions. Firstly, the method can aid in the selection of kernel and regularization parameters in order to select parameters that lead to a visualizable SVM model. As such, it offers a way to (possibly) explain SVM models, but at this stage, it is not completely clear why certain models cannot be represented in this way. When the SVM model can be explained, a second question comes up: How should the different components be interpreted? This will be investigated in future research but the claim that components with small effects can always be ignored and components with large effects are always important is certainly not true. It is well possible that a main effect and an interaction effect counter-balance each other. Assume a linear main effect of input *x*^(3)^ and an interaction effect between *x*^(3)^ and *x*^(4)^. It is possible that the modelled interaction effect in fact barely depends on *x*^(4)^ and that the interaction is actually a main effect of *x*^(3)^. If both this effect and the estimated main effect of *x*^(3)^ are each others opposite, there is in fact no effect of *x*^(3)^. It is therefore very important to investigate the modelled effects together with their range and interpret the results carefully. This issue is a result of the non-unique nature of additive models involving main and interaction effects. Whether this can be solved by means of other, more restrictive expansions of the RBF kernel is a topic for future research.

Another aspect involves the representation of the approximation. The chosen representation resembles nomograms for standard statistical risk prediction models, with the advantages of being able to (i) represent non-linearities without the need for repetitive scales and (ii) represent interactions between continuous inputs in a continuous way. The color in this representation offers the same interpretation as the length of the scales for nomograms. Both methods suffer from outliers. When one input has an outlier, this is not visible in the representation provided by nomograms, nor the representation provided here. The code for the presented representation however enables to include lines to indicate the fifth and ninetyfifth percentile of the training data on top of the color bars for continuous inputs such that it can be indicated whether the data are skewed or outliers are present. The code also provides a way to alter the representation by adapting the range of the input variables, as such allowing to reduce the impact of outliers on the visualization.

Similarly to the length of the bars for standard nomograms, the range of the colors indicates the importance of the input variables. However, applying automated feature selection based on this range (as proposed in [40]) might not be ideal. The range should be combined with a distribution of the input variables, since an outlier might have a large effect on the color representation, but should not have a large effect when performing feature selection. When taking the distribution of inputs into account, the visualization can be used to select relevant contributions. Based on the selected set of contributions, ANOVA kernels [41] containing only these terms could be used to train a sparser, data-specific kernel. We stress however, that this will only yield satisfactory results when (i) the visualization is the result of a well performing approximation of the SVM model, (ii) the effect of outliers is not taken into account and (iii) correlations between the contributions have been investigated to identify irrelevant input variables.

An obvious disadvantage of the approach is the exponential increase in the number of interaction plots in the representation with increasing dimension of the data set. This hampers the use of the presented methodology for data sets with even moderate dimensionality.

The methodology elaborated on in this work is not restricted to SVM classification nor to the kernels discussed here. Extensions towards SVM regression, least-squares support vector machines [42, 43] and other kernel-based methods should be straightforward. An example is that the projection of a new input on each non-linear principal component of kernel principal component analysis [44], could be explained by contributions from the original input space. Experiments will indicate whether the rest term can be ignored in this case.

The proposed technology also offers possibilities in other domains. The developed applications can be used for teaching purposes since it is ideal to illustrate the working of different kernels within machine learning methods. The tools can also aid in the communication with end users of classifiers. They offer a way to increase the credability of black-box models, since experts can compare the working of the classifier with domain knowledge. It can also aid in the communication of risk to patients by indicating which inputs are related to a certain diagnosis or prognosis and might as such explain treatment choices and improve therapy adherence.

When an SVM model can be explained using the proposed methodology and the working of the model is counterintuitive, in contrast to expert knowledge or too complex to be realistic, the model can be rejected for practical applications, although test performance might be high. As such, the methods are also able to provide evidence why not to use SVM models.

## Conclusions

This work proposes to approximate an SVM model by a sum of main and two-way interaction terms that can be visualized, to illustrate and explain SVM models. It is shown that in some cases the approximation is exact. In other cases a rest term is ignored and interpretation of the results is only possible whenever this rest term is small.

The proposed method should not be considered as a final way to explain and visualize the working of SVM models. It is one step that enables explanation in certain cases, but cannot solve the issues of black box models completely. However, it offers the opportunity to open debate on the use, application and interpretation of SVMs in areas where interpretability is important. End users now have a tool that provides them with the possibility to compare the SVM model with expert knowledge, providing evidence (or not) of how the model obtains a class label or risk prediction.

## Supporting Information

S1 Video Application of the method to the IRIS data set. This video illustrates the possibilities of the R package by means of an R application using the IRIS data set.
(MP4)

S1 Text Explanation of how a color based nomogram results from a risk prediction model. This appendix explains in detail how a risk prediction model that can be represented by means of Eq (3) can be represented by the proposed color based nomogram.
(PDF)

S2 Text Definition of the different contributions in the approximation of the SVM classifiers. This appendix summarizes how the terms *f*^(*q*)^ and *f*^(*p*,*q*)^ used in the expansion of the SVM model, are calculated for the linear, polynomial and RBF kernel.
(PDF)

S3 Text Setting of artificial examples. This appendix illustrates the settings of the artificial examples.
(PDF)

S1 Fig Bivariate plot of the Iris data (training data). (PDF)
