# Supplementary material for: Explaining Support Vector Machines: A Color Based Nomogram
Source: PLoS One. 2016 Oct 10;11(10):e0164568. doi: 10.1371/journal.pone.0164568 (PMC5056733; doi:10.1371/journal.pone.0164568)

Explanation of how a color based nomogram results from a risk prediction model.

The visualization of a risk prediction model as a color based nomogram is based on the formulation of the risk prediction model in the following form:

$$\hat{y}(x) = h\left(\sum_{p=1}^{d} f^{(p)}(x^{(p)})\right).$$

The Figure below illustrates the links between the different parts of this formula and the plot. For each input $x^{(p)}$ a color bar represents the impact $f^{(p)}$ of the value of this input onto the linear predictor or latent variable. The sum of all these contributions for one particular observation is called the score and is related to the linear predictor or latent variable. The difference is that the score can be a translated version of the latter: the constant term is not included, and the minimum or median value of a contribution might be substracted from it. The function $h(\cdot)$, converting the score into a risk prediction is represented by means of the bottom most color bar. When representing logistic regression models, this function is the sigmoid funcion. For the applications in this work, the sigmoid function with two parameters that need to be tuned was used (Platt's method).

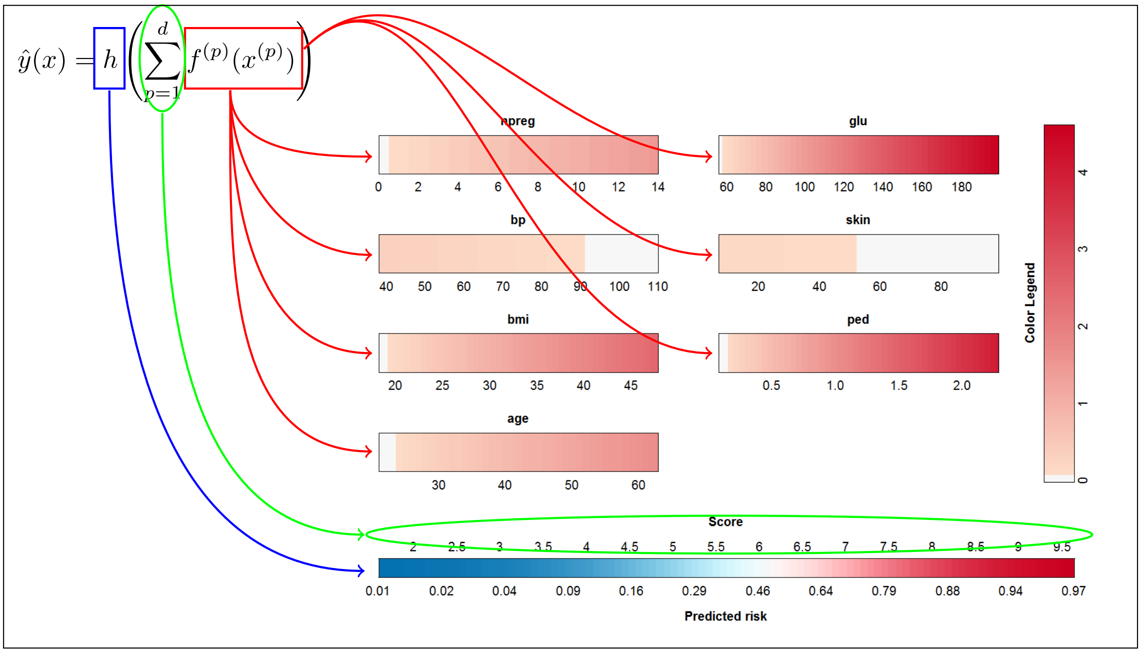

Supplement: S1 Text — This appendix explains in detail how a risk prediction model that can be represented by means of Eq (3) can be represented by the proposed color based nomogram. (PDF) [file pone.0164568.s002.pdf]
