# Supplementary material for: Explaining Support Vector Machines: A Color Based Nomogram
Source: PLoS One. 2016 Oct 10;11(10):e0164568. doi: 10.1371/journal.pone.0164568 (PMC5056733; doi:10.1371/journal.pone.0164568)
Supplement: S2 Text — This appendix summarizes how the terms f(q) and f(p,q) used in the expansion of the SVM model, are calculated for the linear, polynomial and RBF kernel. (PDF) [file pone.0164568.s003.pdf]

Definition of the different contributions in the approximation of the SVM classifiers.

This appendix summarizes how the contributions $f^{(p)}$ and $f^{(p,q)}$ to the latent variable can be calculated for the linear, polynomial and RBF kernel.

1 Linear kernel

For the linear kernel, contributions involving more than two inputs do not exist, so $f^{(p,q)}$ should not be defined. Contributions involving only one input are defined in the main paper as:

$$f^{(p)} = \sum_{i=1}^{N} \alpha_i y_i x_i^{(p)} x^{(p)} .$$

2 Polynomial kernel

The expansion of the polynomial kernel with $a = 1$ equals:

$$\begin{aligned}
K_{\text{poly}}(x_i, x) &= (x_i^T x + c)^\delta = \left(\sum_{p=1}^{d} x_i^{(p)} x^{(p)} + c \right)^\delta \\
&= c^\delta + \sum_{p=1}^{d} {x_i^{(p)}}^\delta {x^{(p)}}^\delta + \\
&\quad \sum_{p=1}^{d} \sum_{\substack{k_p + k_c = \delta \\ k_p, k_c \neq \delta}} \begin{pmatrix} \delta \\ k_p, k_c \end{pmatrix} {x_i^{(p)}}^{k_p} {x^{(p)}}^{k_p} c^{k_c} + \\
&\quad \sum_{p=1}^{d} \sum_{q \neq p} \sum_{\substack{k_p + k_q = \delta \\ k_p, k_q \neq \delta}} \begin{pmatrix} \delta \\ k_p, k_q \end{pmatrix} {x_i^{(p)}}^{k_p} {x^{(p)}}^{k_p} {x_i^{(q)}}^{k_q} {x^{(q)}}^{k_q} + \\
&\quad \sum_{p=1}^{d} \sum_{q \neq p} \sum_{\substack{k_p + k_q + k_c = \delta \\ k_p, k_q, k_c \neq \delta \\ k_p + k_q \neq \delta \\ k_p + k_c \neq \delta \\ k_q + k_c \neq \delta}} \begin{pmatrix} \delta \\ k_p, k_q, k_c \end{pmatrix} {x_i^{(p)}}^{k_p} {x^{(p)}}^{k_p} {x_i^{(q)}}^{k_q} {x^{(q)}}^{k_q} c^{k_c} + \Delta \\
&= \sum_{p=1}^{d} g^{(p)} \left(x_i^{(p)}, x^{(p)} \right) + \sum_{p=1}^{d} \sum_{q \neq p} g^{(p,q)} \left(x_i^{(p,q)}, x^{(p,q)} \right) + b' + \Delta .
\end{aligned}$$

The kernel is hereby decomposed in terms $g^{(p)}$ that solely depend on the pth input variable:

$$
\begin{aligned}
g^{(p)}\left(x_i^{(p)}, x^{(p)}\right) &= \left(x_i^{(p)} x^{(p)}\right)^{\delta} + \sum_{\substack{k_p + k_c = \delta \\ k_p, k_c \neq \delta}} \begin{pmatrix} \delta \\ k_p, k_c \end{pmatrix} {x_i^{(p)}}^{k_p} {x^{(p)}}^{k_p} c^{k_c} \\
&= K_{\text{poly}}(x_i^{(p)}, x^{(p)}) - c^{\delta} \,,
\end{aligned}
$$

and terms that depend on two input variables $x^{(p)}$ and $x^{(q)}$:

$$
\begin{aligned}
g^{(p,q)}\left(x_i^{(p,q)}, x^{(p,q)}\right) &= \sum_{\substack{k_p + k_q = \delta \\ k_p, k_q \neq \delta}} \begin{pmatrix} \delta \\ k_p, k_q \end{pmatrix} \left(x_i^{(p)} x^{(p)}\right)^{k_p} \left(x_i^{(q)} x^{(q)}\right)^{k_q} + \\
&\quad \sum_{\substack{k_p + k_q + k_c = \delta \\ k_p, k_q, k_c \neq \delta \\ k_p + k_q \neq \delta \\ k_p + k_c \neq \delta \\ k_q + k_c \neq \delta}} \begin{pmatrix} \delta \\ k_p, k_q, k_c \end{pmatrix} {x_i^{(p)}}^{k_p} {x^{(p)}}^{k_p} {x_i^{(q)}}^{k_q} {x^{(q)}}^{k_q} c^{k_c} \\
&= K_{\text{poly}}(x_i^{(p,q)}, x^{(p,q)}) - g^{(p)}\left(x_i^{(p)}, x^{(p)}\right) - g^{(q)}\left(x_i^{(q)}, x^{(q)}\right) - c^{\delta} \,.
\end{aligned}
$$

The latent variable of the SVM model using this kernel can then be decomposed in terms $f^{(p)}$ depending on the pth input variable:

$$
f^{(p)}\left(x_i^{(p)}, x^{(p)}\right) = \sum_{i=1}^{N} \alpha_i y_i g^{(p)}\left(x_i^{(p)}, x^{(p)}\right)
$$

and terms depending on two input input variables:

$$
f^{(p,q)}\left(x_i^{(p,q)}, x^{(p,q)}\right) = \sum_{i=1}^{N} \alpha_i y_i g^{(p,q)}\left(x_i^{(p,q)}, x^{(p,q)}\right).
$$

3 RBF kernel

The RBF kernel can be expanded as:

$$
\begin{aligned}
K_{\text{RBF}}(x_i, x) &= \sum_{n=0}^{\infty} \frac{(-1)^n \gamma^n}{n!} \left[\sum_{p=1}^{d} (x_i^{(p)} - x^{(p)})^{2n} + \right. \\
&\quad \left. \sum_{p=1}^{d} \sum_{q \neq p} \sum_{\substack{k_p + k_q = n \\ k_p, k_q \neq n}} \begin{pmatrix} n \\ k_p, k_q \end{pmatrix} (x_i^{(p)} - x^{(p)})^{2k_p} (x_i^{(q)} - x^{(q)})^{2k_q} \right] \\
&\quad + \Delta \,, \\
&= \sum_{p=1}^{d} g^{(p)}\left(x_i^{(p)}, x^{(p)}\right) + \sum_{p=1}^{d} \sum_{q \neq p} g^{(p,q)}\left(x_i^{(p,q)}, x^{(p,q)}\right) + \Delta \,.
\end{aligned}
$$

The kernel is hereby decomposed in terms $g^{(p)}$ that solely depend on the pth input variable:

$$
\begin{aligned}
g^{(p)}\left(x_i^{(p)}, x^{(p)}\right) &= \textstyle\sum_{n=0}^{\infty} \frac{(-1)^n \gamma^n}{n!} \sum_{p=1}^{d} (x_i^{(p)} - x^{(p)})^{2n} \\
&= K_{\text{RBF}}(x_i^{(p)}, x^{(p)}) \,,
\end{aligned}
$$

and terms that depend on two input variables $x^{(p)}$ and $x^{(q)}$:

$$
\begin{aligned}
g^{(p,q)}\left(x_i^{(p,q)}, x^{(p,q)}\right) &= \textstyle\sum_{n=0}^{\infty} \frac{(-1)^n \gamma^n}{n!} \displaystyle\sum_{\substack{k_p + k_q = n \\ k_p, k_q \neq n}} \begin{pmatrix} n \\ k_p, k_q \end{pmatrix} (x_i^{(p)} - x^{(p)})^{2k_p} (x_i^{(q)} - x^{(q)})^{2k_q} \\
&= K_{\text{RBF}}(x_i^{(p,q)}, x^{(p,q)}) - K_{\text{RBF}}(x_i^{(p)}, x^{(p)}) - K_{\text{RBF}}(x_i^{(q)}, x^{(q)}) \,.
\end{aligned}
$$

The latent variable of the SVM model using this kernel can then be decomposed in terms $f^{(p)}$ depending on the pth input variable:

$$f^{(p)}\left(x_i^{(p)}, x^{(p)}\right) = \sum_{i=1}^{N} \alpha_i y_i g^{(p)}\left(x_i^{(p)}, x^{(p)}\right)$$

and term depending on two input input variables:

$$f^{(p,q)}\left(x_i^{(p,q)}, x^{(p,q)}\right) = \sum_{i=1}^{N} \alpha_i y_i g^{(p,q)}\left(x_i^{(p,q)}, x^{(p,q)}\right).$$
