# Supplementary material for: Explaining Support Vector Machines: A Color Based Nomogram
Source: PLoS One. 2016 Oct 10;11(10):e0164568. doi: 10.1371/journal.pone.0164568 (PMC5056733; doi:10.1371/journal.pone.0164568)
Supplement: S3 Text — This appendix illustrates the settings of the artificial examples. (PDF) [file pone.0164568.s004.pdf]

Setting of artificial examples.

Figure 1 illustrates the problem settings of the artificial experiments performed in the main manuscript.

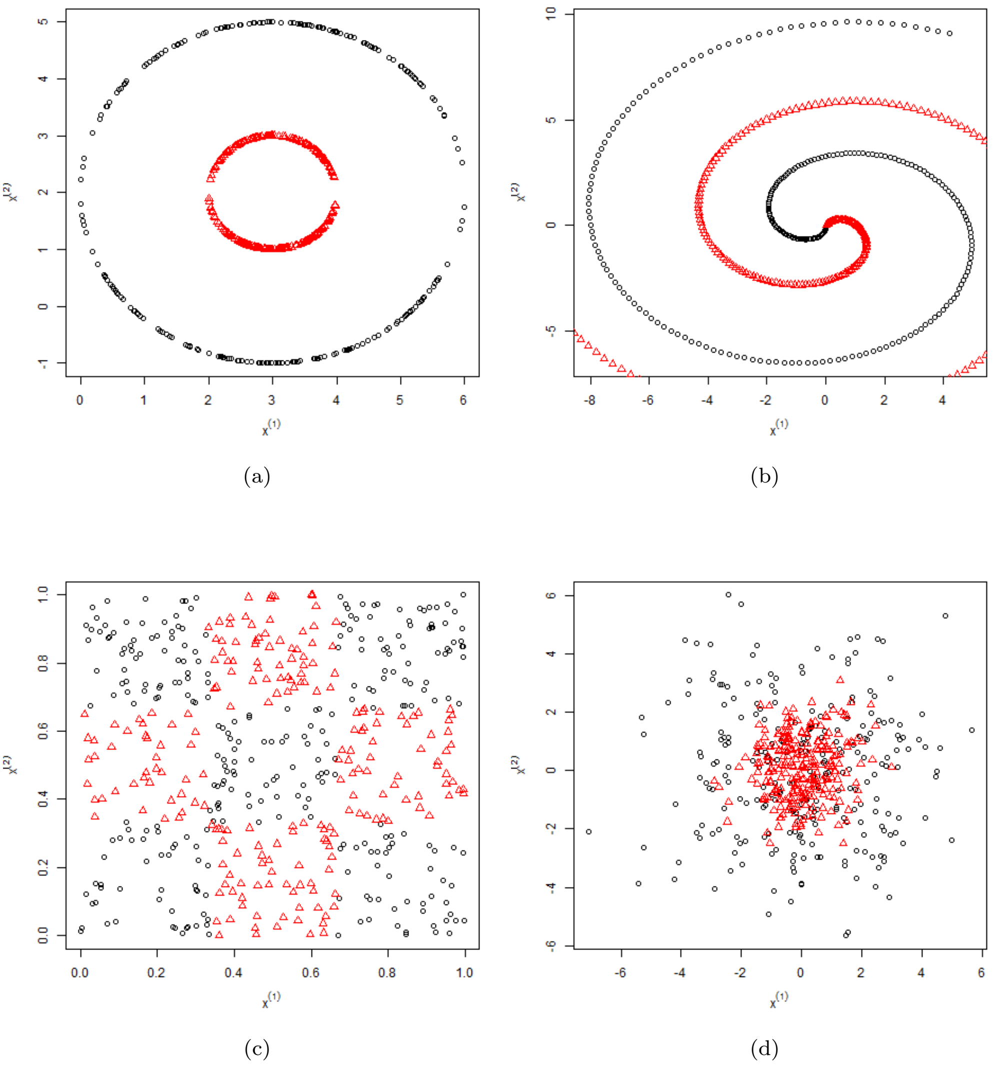

Figure 1: Visualization of the artificial datasets in a two-dimensional space, spanned by the two relevant input variables $x^{(1)}$ and $x^{(2)}$. Observations of the first class are indicated by black circles, observations of the second class by red triangles. (a) two-circles, (b) swiss roll, (c) xor, (d) two Gaussians.
