# Supplementary figures and images for: Explaining Support Vector Machines: A Color Based Nomogram
Source: PLoS One. 2016 Oct 10;11(10):e0164568. doi: 10.1371/journal.pone.0164568 (PMC5056733; doi:10.1371/journal.pone.0164568)

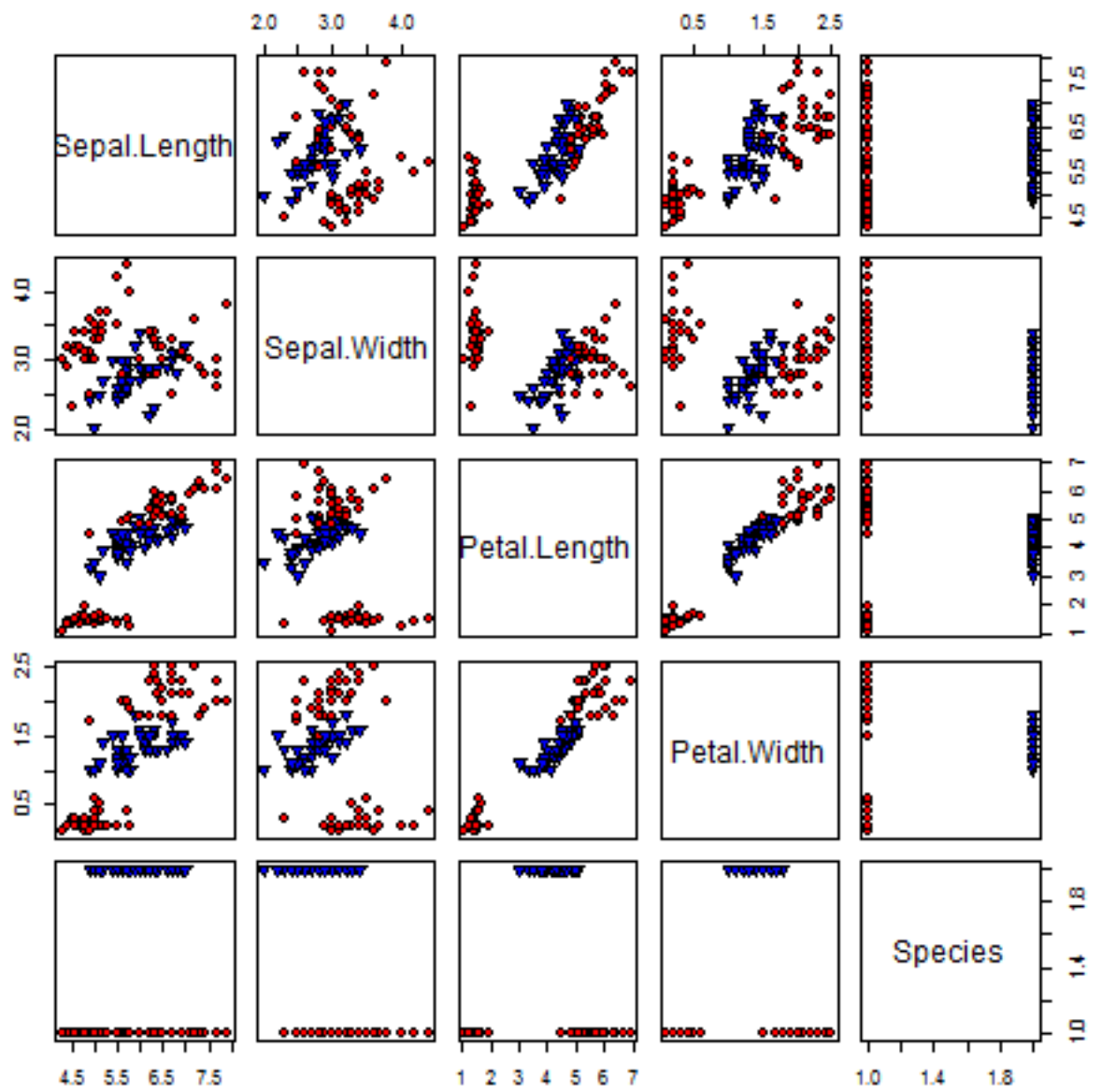

Figure 1: Bivariate plot of the Iris data (training data).

Supplement: S1 Fig — (PDF) [file pone.0164568.s005.pdf]
